# Genome-Wide Identification of the Physic Nut WUSCHEL-Related Homeobox Gene Family and Functional Analysis of the Abiotic Stress Responsive Gene *JcWOX5*

**DOI:** 10.3389/fgene.2020.00670

**Published:** 2020-06-19

**Authors:** Yuehui Tang, Han Li, Yaxin Guan, Shen Li, Chunfei Xun, Yanyang Dong, Rui Huo, Yuxi Guo, Xinxin Bao, Enqing Pei, Qianmiao Shen, He Zhou, Jingjing Liao

**Affiliations:** ^1^Key Laboratory of Plant Genetics and Molecular Breeding, Zhoukou Normal University, Zhoukou, China; ^2^Henan Key Laboratory of Crop Molecular Breeding and Bioreactor, Zhoukou, China; ^3^School of Journalism and Communication, Zhoukou Normal University, Zhoukou, China

**Keywords:** WOX gene family, physic nut, expression profile, *JcWOX5*, transgenic rice

## Abstract

Plant-specific WOX transcription factors have important regulatory functions in plant development and response to abiotic stress. However, the identification and functional analysis of members of the WOX family have rarely been reported in the physic nut plant until now. Our research identified 12 *WOX* genes (*JcWOXs*) in physic nut, and these genes were divided into three groups corresponding to the ancient clade, WUS clade, and intermediate clade. Expression analysis based on RNA-seq and qRT-PCR showed that most of the *JcWOX* genes were expressed in at least one of the tissues tested, whereas five genes were identified as being highly responsive to drought and salt stresses. Subcellular localization analysis in *Arabidopsis* protoplast cells showed that *JcWOX5* encoded a nuclear-localized protein. *JcWOX5*-overexpression plants increased sensitivity to drought stress, and transgenic plants suggested a lower proline content and CAT activity, higher relative electrolyte leakage, higher MDA content, and higher rate of water loss under drought conditions. Expression of some stress-related genes was obviously lower in the transformed rice lines as compared to their expression in wild-type rice lines under drought stress. Further data on *JcWOX5*-overexpressing plants reducing drought tolerance verified the potential role of *JcWOX* genes in responsive to abiotic stress. Collectively, the study provides a foundation for further functional analysis of *JcWOX* genes and the improvement of physic nut crops.

## Introduction

Environmental factors, particularly salt and drought stress, severely limit the production and distribution of many important agronomic crops worldwide. To survive external extreme environment stress, plants have evolved multiple mechanisms as a defense strategy against external signals by modulating the expression of genes. Among these genes, transcription factors, such as HD-Zip, WOX, ERF, WRKY, NAC, and MYB, have clearly played a key role in regulating plant response to drought or salt stress (Butt et al., 2017; Jiang et al., 2017; Tang et al., 2019b; Xie et al., 2019; Yuan et al., 2019).

Members of the WOX gene family encode proteins that are unique to plants, and belongs to the subclades of the homeobox (HB) superfamily. WOX transcription factors have been shown to usually contain a highly conserved homeodomain (HD) domain responsible for binding to specific DNA, and the HD domain contains an HLHTH (helix-loop-helix-turn-helix) structure of about 60–66 amino acid residues (Yang et al., 2017). In addition, the HLHTH structure is essential for the HD to perform its function, and the role played by the domain is extremely important (van der Graaff et al., 2009). Based on the conserved domain and similarity of full-length amino acid sequence, the researchers divided the WOX proteins into three groups, namely intermediate clade, ancient clade, and WUS clade (van der Graaff et al., 2009).

Members of the WOX gene family have been comprehensively identified or predicted in many plants, such as *Arabidopsis*, maize, soybean, rice etc. (van der Graaff et al., 2009; Zhang et al., 2010; Hao et al., 2019). Plant *WOX* genes have been found to be involved in diverse biological and physiological processes in regulating plant development (Ueda et al., 2011; Costanzo et al., 2014). For example, *STF* (a WOX family transcription factor) overexpressing switchgrass plants exhibit an increased biomass yield and sugar release (Wang et al., 2017). In poplar, *PeWOX11a* and *PeWOX11b* are found to have important roles in regulating root, axillary bud, and leaf development (Xu et al., 2015). In rice, overexpression of NAL2/3 results in wider leaves in transgenic plants (Ishiwata et al., 2013). *RcWOX1*-overexpressing *Arabidopsis* plants enhance lateral root density by regulating the expression of *PIN1* and *PIN7* genes (Gao et al., 2014). In cotton, *WOX13* is determined to be an essential regulator in fiber development (He et al., 2019). *PpWOX13L* has been confirmed to function as a regulator during the initiation of stem cell growth (Sakakibara et al., 2014). *WOX8* and *WOX9* have key regulatory roles in the embryo axis formation (Ueda et al., 2011), and the role played by *WOX13* mainly involves the development of *Arabidopsis* replum (Romera-Branchat et al., 2013). In *Arabidopsis*, *AtWOX6*/*PSF2* has a key regulatory role in ovule development by medicating cell proliferation (Park et al., 2005), while the role played by *WOX5* is to participate in the regulation of a correct root-formation pattern (Gonzali et al., 2005). In addition, evidence is accumulating to suggest that *WOX* genes also participate in the regulation of abiotic stress resistance. For example, in rice, overexpression of *OsWOX13* under the *rab21* promoter increases drought stress tolerance (Minh-Thu et al., 2018). In *Arabidopsis*, *HOS9* demonstrates that it can regulate cold stress tolerance (Zhu et al., 2004). However, although various members of the WOX family have been cloned and functionally studied, little is known about the members of this family and their roles in many taxa, or even plants from the Euphorbiaceae.

*Jatropha curcas* is one of the most important non-edible Euphorbiaceae crop, used for biofuel, feed, and a range of industrial applications worldwide owing to its high seed oil content, rapid growth, ease of propagation, and extensive adaptability (Openshaw, 2000). Therefore, *WOX* genes have been identified as potential targets for better, faster, and more stress-resistant growth of physic nut due to the key regulatory role played by these genes in growth and development. Based on this, we firstly searched for and identified 12 *WOX* genes in *J. curcas* (hereafter *JcWOX* genes). Next, we provided a detailed analysis of the phylogenetic, gene structure, conserved motifs, and expression profile of the identified *JcWOX* genes. Finally, we detected the function of a drought stress-responsive gene *JcWOX5* in rice. Our research will provide a good foundation for further research on the potential function of *JcWOX* genes involved in the regulation of physic nut growth and development, and abiotic stress.

## Materials and Methods

### Plant Materials

The material used in our research was inbred cultivar GZQX0401 from *J. curcas*, because the genome sequencing of the tree species has been completed and released (Wu et al., 2015), while the other material was the japonica rice (*Oryza sativa* L.) cv. Zhonghua 11 (ZH11).

### Identification of *JcWOX* Genes in Physic Nut

The HMM profile of the HD domain (PF00046) was used to do a BLASTP search in the physic nut genome database with an *e*-value cut-off of 0.01. In addition, all WOX proteins from *Arabidopsis* were used as query to perform BLASTP against the genome database from physic nut. All putative *JcWOX* sequences were collected and the redundant sequences were manually removed; the remaining candidate JcWOX proteins were submitted to a SMART (ID was SM000389), Pfam (ID was PF00046), and NCBI Conserved Domain Database (CDD) (ID was PF00046) search to confirm the existence of the HD domain. At this time, we removed these sequences that did not contain the HD conserved domain.

### Phylogenetic and Gene Structure Analysis of *WOX* Genes

The *Arabidopsis* genome sequence database^1^ was employed to download *Arabidopsis* WOX protein, and for rice WOX proteins, the Phytozome^2^ was used to download these proteins. Sequences for poplar, soybean, and *J. curcas* were from the GenBank database^3^. Multiple alignments were analyzed using the Clustal X software (1.83). Phylogenetic trees were built according to the following parameters: NJ (neighbor-joining) method, 1000 bootstraps, and the software was MEGA 6. We hired GSDS^4^ to analyze gene structure.

### Conserved Motif, Amino Acid Sequence, and Chromosome Location Analysis of *JcWOX* Genes

MEME (Version 5.1.0) was used to analysis the conserved motif of JcWOX proteins. MEME was run online with the following requirements: site distribution (zero or one occurrence per sequence), motif width (between 6 wide and 100 wide), an order-0 background, and motif count (15 motifs). DNAMAN software was used for amino acid sequencing of JcWOX proteins analysis. Data about the chromosome locations of *JcWOX* genes in physic nut genomes was retrieved from the physic nut database, and MapChart software was used to draft their positions to LGs.

### Expression Profile Analysis of *JcWOX* Genes

Our research used the roots, stem cortexes, leaves, and seeds from days 14 and 35 of 21-day-old *J. curcas* seedlings for RNA-seq analysis. Regarding salt stress, 21-day-old *J. curcas* seedlings were directly watered with nutrient solution (Hoagland) containing 100 mM NaCl, however, regarding drought stress, watering was stopped directly. And then roots 2, 4, and 7 days after drought stress, and 2 h, 2, and 4 days after salt stress were collected and used for RNA-seq analysis. Regarding qRT-PCR, for drought stress, 3-week-old *J. curcas* seedlings were directly watered with nutrient solution (Hoagland) containing 20% PEG6000, and then roots at 0, 3, 6, and 12 h after drought and salt stress were used for qRT-PCR analysis. The available number of drought stress raw data for the SRV (sequence read archive) at NCBI was PRJNA257901, whereas salt stress was PRJNA244896.

### Subcellular Localization of *JcWOX5* Gene

The full length CDS sequence (without stop codon) of *JcWOX5* was obtained via RT-PCR. The PCR product was ligated to the pSAT6-eYFP-N1 plasmid by T4 DNA ligase, and then a fusion expression vector from 35S:JcWOX5-YFP was generated. Subsequently, we transferred the 35S:JcWOX5-YFP and 35S:YFP plasmids into protoplast cells from *Arabidopsis* by PEG-mediate method. Finally, we observed the YFP fluorescence under the fluorescence confocal microscope equipped with LSM Image Browser software. *Arabidopsis* protoplasts were prepared following Abel and Theologis (1998).

### Gene Cloning and Plant Transformation

RT-PCR was used to clone the coding region sequence of *JcWOX5* gene using the total RNA of physic nut roots as template. After recovering the amplified sequence of the *JcWOX5* gene, the gene to the pMD18-T vector was connected. After confirmation of its identity by DNA sequencing, the target sequence was excised from the pMD18-T vector after digestion with *Kpn*I and *Xba*I. Next, the gene was connected to the plant expression vector pCAMBIA1301 by T4 DNA ligase to construct a plant expression vector in which the CaMV 35S promoter controlled the expression of the *JcWOX5* gene.

The constructed *JcWOX5* expression vector was transferred into the EHA105 strain via the freeze–thaw procedure. Subsequently, the transformation solution containing the EHA105 strain was used to transform and produce rice transgenic plants, as described by Tang et al. (2019b).

### Stress Treatment and the Rate of Water Loss Analysis

For drought stress, seedlings of approximately 3 cm wild-type and *JcWOX5* overexpressing plants with a uniform growth were selected and planted in round pots containing nutrient soil and vermiculite (1:3) at 28°C (day/night) with a 12 h photoperiod, during which time they were uniformly watered with rice nutrient solution. After 2 weeks, watering was stopped for drought stress treatment for 20 days. The seedlings were then immediately rehydrated for 5 days. The experiment contained three biological replicates, and each replicate had similar results.

To examine the rate of water loss in response to drought stress, the leaves of 21-day-old WT (wild-type) and *JcWOX5*-overexpressing plants were cut off, and then weighed immediately as the initial weight under normal growth conditions. These isolated leaf samples were placed on the platform of the growth chamber at 28°C and weighed every hour. According to the leave’s first weight, the water loss rate was statistically analyzed.

### Physiological Indices Analysis

The leaves treated with drought stress for 12 days were used to detect relative electrolyte leakage (REL), proline and MDA contents, and CAT activity. For REL detection, the leaves were first cut into a 0.5 cm shape, then washed 6 times with deionized water, and then put into a test tube containing 10 mL of deionized water. Next, the test tube was continuously shaken on the oscillator for 3 h, at which time the conductivity (C1) of the solution was measured with a conductivity meter. Subsequently, the test tube was placed in the boiler for 20 min, and after waiting for it to cool to room temperature, the conductivity (C2) was detected again through the conductivity meter. The relative conductivity was calculated by the following formula: REL (%) = C1/C2 × 100.

For MDA measurement, the leaves were first put in the mortar and 5% (w/v) trichloroacetic acid was added, followed by continuous grinding for 3 min. Then 0.67% (w/v) thiobarbituric acid was added, and reacted for 30 min. After cooling to room temperature, three different wavelengths (532, 600, and 450) were selected, and the OD value of the supernatant was measured at these three wavelengths. Finally, the content of MDA was calculated by the following formula: 6.45 × (OD532 – OD600) – 0.559 × OD450.

For proline content and CAT activity, the method in our previously reported paper was used to detect the activity of CAT and the content of proline (Tang et al., 2019a).

### RNA Isolation and qRT-PCR Analysis

The method of RNA extraction in this study was as follows. The extraction of total RNA from all materials was carried out by Megan’s plant RNA extraction kit. For specific operations, refer to the kit instructions. The cDNA was synthesized using the PrimeScript IV 1st strand cDNA Synthesis Mix (TAKARA, Beijing, China). qRT-PCR was performed strictly by the Mini Option real-time PCR system (LightCycler 480) under the following conditions: 95°C at 30 s was followed by 95°C at 5 s, then 60°C at 20 s, and then 72°C at 20 s. The reaction was carried out for 40 cycles. To determine relative expression level, we used the 2^–ΔΔCT^ method, and rice *OsUbiquitin* gene and physic nut *JcActin* gene were used for normalization. The primers used can be found in Supplementary Table S1.

### Statistical Analysis

Three biological replicates were performed in our experiments. With reference to the statistical experiment of Duncan, we conducted a statistical analysis of the data designed by the experiment in our study (Duncan, 1955).

## Results

### Identification and Characterization of *JcWOX* Genes

To identify the putative *JcWOX* genes in physic nut, a profile hidden Markov model (HMM) search against physic nut genome protein sequences was carried out using the HD domain (PF00046). In addition, protein sequences of all WOX family members of *Arabidopsis* were also used as search sequences against the physic nut genome database through the online BLASTP program. In total, 12 JcWOX proteins were eventually confirmed in physic nut, by confirming the existence of the HD domain based on CDD, PFam, and SMART database searches. According to their chromosome locations, we named 12 *JcWOX* genes as *JcWOX1* to *JcWOX12*. The CDS sequence of the 12 *JcWOX* genes ranged from 573 bp (*JcWOX11*) to 1233 bp (*JcWOX4*). The deduced JcWOX proteins ranged in length from 190 to 410, while molecular weight varied from 21.7 to 45.2 kDa, and the isoelectric points of these genes ranged from 5.15 to 9.51 (Supplementary Table S2).

### Conserved Amino Acid Sequences Within the Homeodomain Domain

To study the sequence of the conserved HD domains in physic nut, we performed a multi-sequence protein alignment analysis on the amino acid sequences of 12 JcWOX proteins. The detailed sequence analysis suggested that the HD domain of all JcWOX proteins contained three conserved structures, which were loop (1), turn (1), and helix (3), and these structures consisted of 57 amino acids (Supplementary Figure S1). The results also suggested that the amino acids in the loop and turn structures were more variable than those in the helix structure, and helix3 was the most conservative of the three helix structures. In Helix3, seven highly conserved amino acids were found, and they were N, V, W, F, Q, N, and R (Supplementary Figure S1). In conclusion, the HD domain of WOX transcription factor was also very conserved among members of the physic nut JcWOX family.

### Phylogenetic Analysis of WOX Genes

**FIGURE 1:**
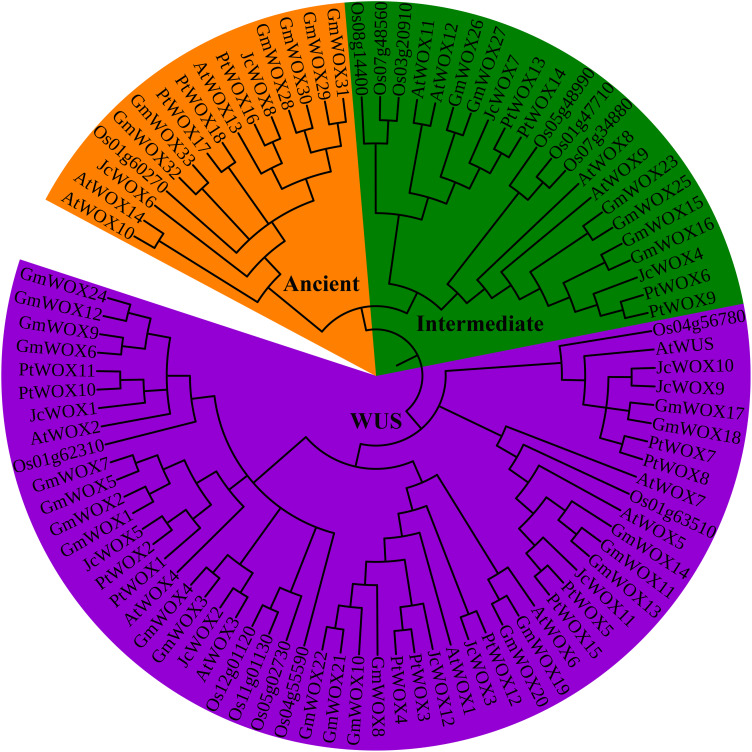
Neighbor-joining unrooted tree of WOX proteins from physic nut, poplar, soybean, and *Arabidopsis*. Bootstrap values were calculated for 1000 replicates, and values are indicated at the corresponding nodes. Different clade genes were marked and grouped in different colors (ancient clade, intermediate clade, and WUS clade was labeled in orange, green, and purple, respectively).

To study the evolutionary relationship of WOX proteins in different species, we constructed a rootless phylogenetic tree with 92 WOX proteins using the neighbor-joining method (of the 92 WOX proteins, as shown in Supplementary Table S3, 15 belonged to *Arabidopsis*, 14 belonged to rice, 12 belonged to physic nut, 33 belonged to soybean, and 18 belonged to poplar). Our results indicated that WOX proteins were dispatched into three groups/clades corresponding to the ancient clade, intermediate clade, and WUS clade (Figure 1). The phylogenetic also showed that the number of WOX transcription factors in the WUS clade (55) was the largest, which was significantly higher than the total number of the intermediate clade (22) and ancient clade (15). Obviously, the WUS clade had the largest number of WOX proteins in these five species.

To further confirm the results of the phylogenetic classification of the WOX protein above, another unrooted phylogenetic tree was constructed using WOX proteins from physic nut and *Arabidopsis*. The results suggested that 27 WOX proteins were classified into three groups: ancient clade, intermediate clade, and WUS clade (Supplementary Figure S2). These results further support the classification of WOX proteins.

### Gene Structure and Conserved Motif Analysis of *JcWOX* Genes

**FIGURE 2:**
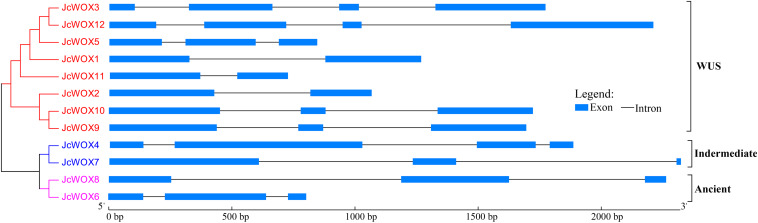
Phylogenetic relationships and gene structure in *WOX* genes from physic nut. Exons and introns are shown as light blue boxes and thin lines, respectively. The unrooted tree was constructed, using the MEGA6.0 program, by the neighbor-joining method. Gene classes are indicated with different colors.

To clarify the evolution of the WOX family in physic nut, we examined the exon-intron structure of all the identified *JcWOX* genes by comparing the corresponding genomic DNA sequences. These results showed that all of the coding sequences of the *JcWOX* genes were disrupted by different numbers of introns (Figure 2). For example, *JcWOX2* and *11* only contained one intron, while *JcWOX3* and *12* contained the largest number of introns (3). The remaining *JcWOX* genes contained two introns.

We further analyzed conserved motifs of all JcWOX proteins using the MEME motif analysis website. As shown in Supplementary Figure S3, 15 conserved motifs were found in 12 JcWOX proteins, which were designated as motifs 1 to 15. Our results showed that all predicted JcWOX proteins contained motifs 1 and 2 (corresponding to HD domain). It was clear that JcWOX members from the same clades were commonly observed to harbor a similar conserved motif (Supplementary Figure S3). For instance, motifs 5 and 13 were unique to the ancient clade, whereas motif 12 was specific to the intermediate clade. Motifs 3, 6, 7, 9, 10, and 14 were only found in the WUS clade. The similar motif arrangements shared by proteins in the same clade indicated that the structure of these proteins in the same clade was very conservative. Except for the HB domain, the function of most of these conserved motifs was unclear and remain to be elucidated. In short, the conserved motifs and similar exon-intron structures of the JcWOX members from the same clade, together with the phylogenetic analysis results, could provide strong evidence for the reliability of phylogenetic tree classification.

### Chromosomal Localization of *JcWOX* Genes

**FIGURE 3:**
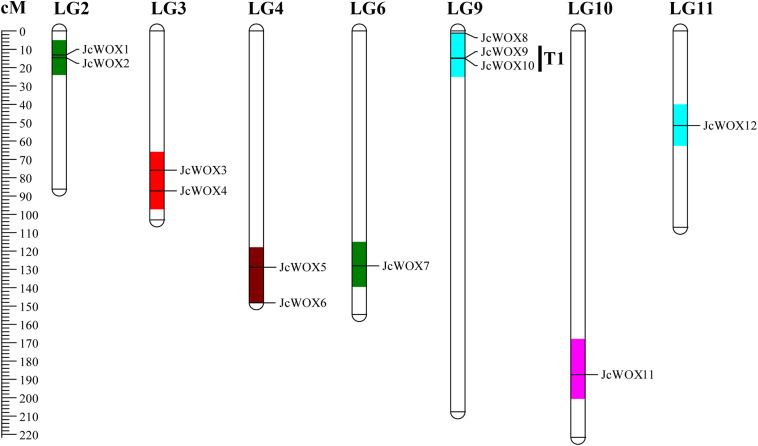
Schematic representations for the chromosomal distribution of physic nut *WOX* genes. The scale is in centiMorgans (cM).

We mapped 12 *JcWOX* genes mapped to LGs using previously published information (Wu et al., 2015). Results suggested that 12 *JcWOX* genes could be located on 7 out of 11 LGs, except for LGs 1, 5, 7, 8, with an obviously non-uniform distribution (Figure 3). LG9 contained the largest number of *JcWOX* genes (3 members). In addition, two *JcWOX* genes were present on LGs 2, 3, and 4, one on LGs 6, 10, and 11. Chromosomal regions are separated by <4 non-homologous spacer or are located within 50 kb from other genes which were defined as tandem duplication events (Cannon et al., 2004). Our results indicated that tandem duplication was found among these members of the *JcWOX* gene family. This tandem repeats gene pair was named T1 (*JcWOX9* and *10*) in the chromosome map.

### Expression Profile of *JcWOX* Genes

**FIGURE 4:**
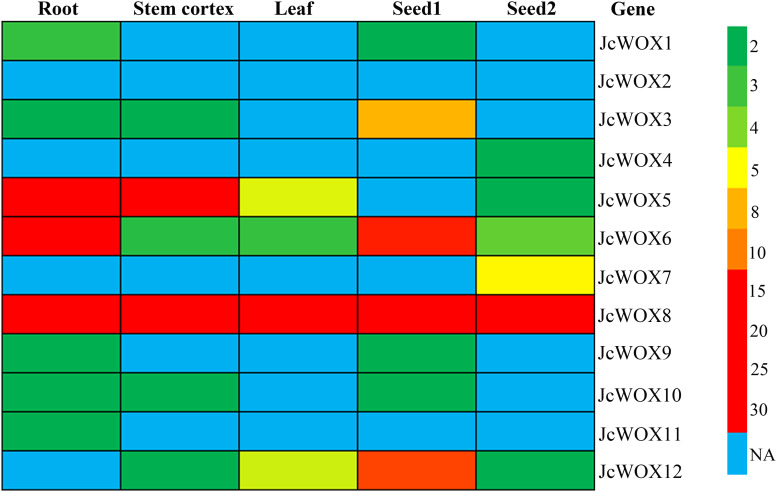
Expression profiles of *JcWOX5*. Patterns of expression of each *JcWOX* gene in physic nut roots, stem cortex, leaves, and seeds at an early developmental stage (S1) and filling stage (S2), with a colored scale indicating expression levels shown on the right.

Expression pattern analysis can provide valuable information for the prediction of gene function. Thus, we examined the expression levels of the 12 *JcWOX* genes based on RNA sequencing data from five samples (roots, stem cortex, leaves, seed 1, and seed 2) (Figure 4 and Supplementary Table S4). The abundance of their transcription was displayed in a heat map. Results suggested that the transcripts of the *JcWOX2* gene were not detected in all organs examined, whereas *JcWOX6* and *8* were expressed in all organs tested. *JcWOX3* and *12* in seed and *JcWOX1* and *5* in root showed the highest transcription abundance.

In addition, as shown in Figure 4, *JcWOX3*, *6*, *8*, and *12* genes had higher transcriptional abundance in the seeds 14 days after pollination (named S1 stage) compared to the transcription of these genes in the seeds 41 days after pollination (named S2 stage). However, *JcWOX7* was found with high expression in the S2 stage, but no expression was detected in the S1 stage. Taken together, the results showed diverse expression levels of *JcWOX* genes in different organs, suggesting that the *JcWOX* genes had multiple functions during physic nut growth and development.

### Expression Profile of *JcWOX* Genes in Abiotic Stress

**FIGURE 5:**
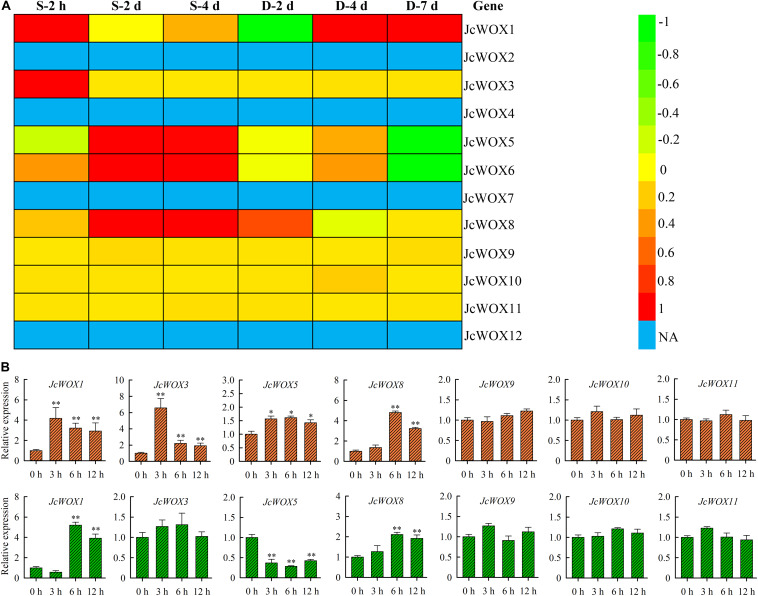
The transcription level of the *JcWOX* gene under drought and salt stress. **(A)** Roots of expression of the 12 *JcWOX* genes in physic nut exposed to drought (D) and salinity (S) stresses: log_2_ ratios of signals from treated versus control leaves are presented as a heat map based on transcriptomic data, with the color scale shown on the right. NA, not available. **(B)** Detection of *JcWOX* genes expression level by qRT-PCR in roots. The experiment contains three biological replicates, and asterisks above the bars indicate significant differences from wild-type controls at *p* < 0.01 (**) or 0.01 < *p* < 0.05 (*).

To gain further insight into the potential roles of physic nut *JcWOX* genes in drought and salinity stresses, we analyzed the transcriptional abundance of *JcWOX* genes in roots in response to salt and drought stresses based on these data from RNA seq. As shown in Figure 5A, 5 *JcWOX* genes suggested differential transcription abundance when faced with at least one abiotic stress treatment from at least one time point. Out of these five detected differential expression *JcWOX* genes, three genes (*JcWOX1*, *5*, and *6*) had undergone an obvious induction or suppression of expression when they encountered drought and salt stresses; two genes (*JcWOX1* and *8*) responded only to salinity stress. We selected an abiotic stress responsive gene (*JcWOX5*) for further analysis.

To confirm the reliability of the RNA-seq results, we further analyzed the expression levels of the *JcWOX* genes in roots when facing drought and salt stress via qRT-PCR (Figure 5B). In our results, the differential expression of *JcWOX* genes tended to be consistent with the data obtained by RNA-seq under drought and salt stress conditions, indicating that our RNA-seq data was very reliable.

### Subcellular Localization of *JcWOX5* Gene

**FIGURE 6:**
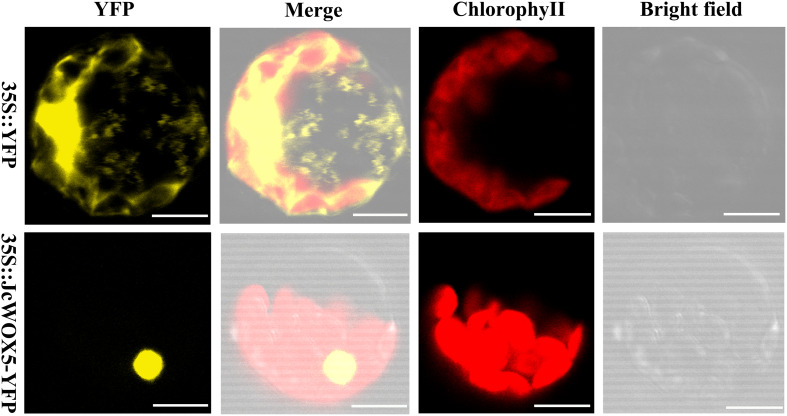
Subcellular localization of *JcWOX5.* The recombinant vector 35S:JcWOX5-YFP and the vector control was individually infiltrated into *Arabidopsis* protoplast cells. The fluorescence was observed under a laser scanning confocal microscopy. Scale bar: 10 μM.

To clarify the subcellular localization of the protein encoded by the *JcWOX5* gene, the sequence of the coding region of the gene after the stop codon was removed was connected to the 5′-terminus of the reporter gene YFP, and the expression of this fusion protein (JcWOX5-YFP) was controlled by the CaMV35S promoter. The constructed 35S:JcWOX5-YFP and empty vector 35S:YFP were introduced into *Arabidopsis* protoplast cells. Our results showed that the fluorescence of JcWOX5-YFP was only detected in the nucleus, whereas that of the control vector was found throughout the cell (Figure 6). Collectively, we conclude that the *JcWOX5* gene is located in the nucleus.

### Phenotypic Analysis of Transgenic Rice Plants Expressing *JcWOX5*

**FIGURE 7:**
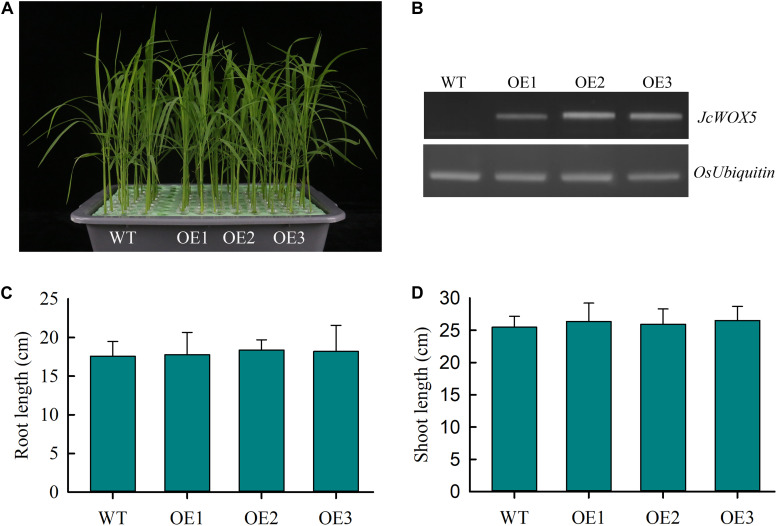
Characterization of *JcWOX5* transgenic plants (OE1, OE2, and OE3) and their growth phenotypes. **(A)** Growth phenotype of 2-week-old wild-type and *JcWOX5* transgenic plants under normal growth conditions. **(B)** Levels of *JcWOX5* transcript in wild-type and transgenic lines. **(C)** Root length in 2-week-old wild-type and transgenic plants. **(D)** Shoot length in 2-week-old wild-type and transgenic plants. Data presented in **(C)** and **(D)** are the means of *n* = 30 ± SD from three independent experiments.

To further elucidate the potential function of *JcWOX5* in plant development and response to abiotic stress, the *JcWOX5* gene was ectopically expressed in rice plants by *Agrobacterium* mediated transformation method. Then, the transcript abundances of *JcWOX5* were detected by RT-PCR, and results showed that the *JcWOX5*-overexpressing plants (OE1, OE2, and OE3) had higher expression levels, but no expression was detected in WT (wild-type) plants (Figure 7B). Our results exhibited that the growth of transgenic crops overexpressing *JcWOX5* was not significantly different from those of WT crops (Figure 7A). Statistical analysis indicated that there was no obvious difference in root and shoot lengths in the transgenic plants compared to the WT plants (Figures 7C,D). Taken together, these results led to the conclusion that *JcWOX5* did not have any obvious impact on the growth of transgenic rice.

### *JcWOX5*-Overexpressing Plants Confers Reduced Drought Tolerance

**FIGURE 8:**
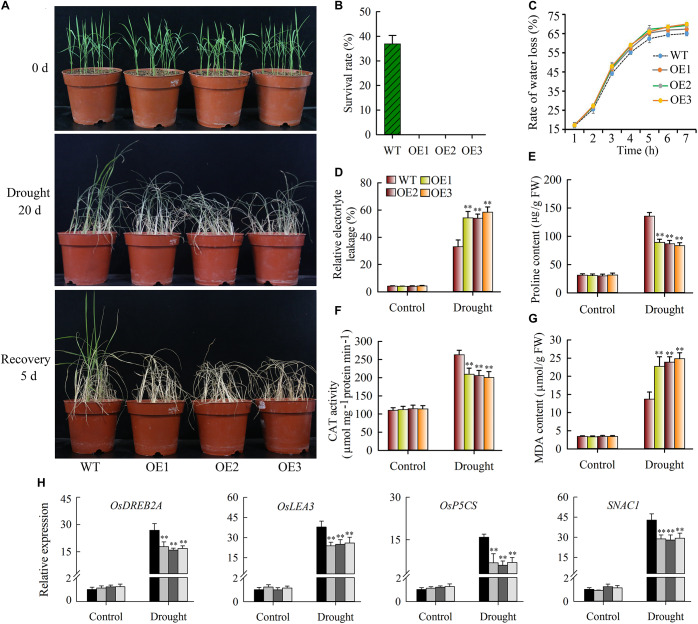
Drought stress tolerance tests on *JcWOX5* overexpressing rice plants. **(A)** Drought stress tolerance tests on *JcWOX5* overexpressing rice lines. Two-week-old seedlings were used for drought stress treatment for 20 days, then rehydrated for 5 days. **(B)** The survival rate of wild-type and transgenic plants after rehydration. **(C)** The rate of water loss in transgenic and wild-type plants under drought stress. **(D)** Relative electrolyte leakage in wild-type and transgenic plants under normal growth and drought stress conditions. **(E)** Proline content in wild-type and transgenic plants under normal growth and drought stress conditions. **(F–G)** CAT activity **(F)** and MDA content **(G)** in leaves before and after drought treatment. Data in **(B–G)**: means of *n* = 20 ± SD from three independent experiments, asterisks above the bars indicate significant differences from wild-type controls at *p* < 0.01 according to Duncan’s multiple range test. **(H)** Relative expression levels of stress-responsive genes, in an experiment with three biological replicates, each with two technical replicates (means of *n* = 6 ± SD, asterisks above the bars indicate significant differences from wild-type controls at *p* < 0.01).

As described above, *JcWOX5* expression was strongly down-regulated by drought stress, suggesting that *JcWOX5* might play significant roles in response to drought stress. Thus, we investigated the effects of drought stress on wild type and *JcWOX5*-overexpressing plants. Our results suggested that the growth of wild-type and transgenic plants exposed to drought stress for 20 days was inhibited, whereas no significant difference in the growth of transgenic and wild-type plants was found under normal conditions (Figure 8A). In addition, the leaf curl and growth inhibition of transgenic plants was significantly higher than that of wild-type (Figure 8A). Obviously, the leaves of wild-type plants were greener than those of transgenic plants after 20 days of drought stress. After 5 days of rehydration, about 35% of the wild-type plants survived, but the leaves of all the transgenic plants appeared curly, whitish, and dead (Figures 8A,B). The water loss rate shared by detached leaves can be used as an indicator to measure the drought resistance of plants. We therefore tested the water loss rate of wild-type and transgenic plants when they encountered drought stress. Our results showed that *JcWOX5-*overexpressing plants had a higher rate of water loss than that of wild type plants (Figure 8C).

Changes in the physiological indices were also evaluated. When the plants were exposed to drought stress, the *JcWOX5*-overexpressing plants suggested significantly higher relative electrolyte leakage and MDA content (Figures 8D,G), but a lower proline content and CAT activity as compared to wild-type plants (Figures 8E,F). These results suggested that the overexpression of *JcWOX5* resulted in physiological changes in transgenic rice, which in turn reduced the observed drought resistance.

### *JcWOX5* Regulates the Expressions of Stress-Responsive Genes Under Drought Stress

To gain further insight into the molecular mechanism underlying the enhanced drought sensitivity in transgenic rice, we tested the level of stress-responsive gene expression in wild-type and transgenic plants when these plants were exposed to drought and non-stress conditions. Our results displayed that some stress-responsive genes, such as *OsLEA3*, *OsSNAC1*, *OsDREB2A*, and *OsP5CS*, had higher transcription abundance in wild-type plants compared to transgenic plants when exposed to drought stress conditions (Figure 8H). However, no obvious difference was detected in wild-type and *JcWOX5* overexpressing plants when exposed to normal conditions (Figure 8H).

## Discussion

The members of the WOX gene family encode plant-specific transcription factors that participated in various biological processes in plants. Up to now, most of the studies on the functions of the *WOX* genes have been focused on the model plants rice and *Arabidopsis* (van der Graaff et al., 2009). The molecular mechanisms involved in response to abiotic stress in the biofuel plant physic nut, and more specifically the identities, expression profiles, and functions of its *WOX* genes, remain poorly understood. We therefore identified and detected expression profiles of *WOX* genes in this species and chose one (we named *JcWOX5*) that inhibited expression in drought stress for further functional analysis by overexpressing it in rice.

In our study, we identified 12 *JcWOX* genes in physic nut. Following the classification of *WOX* genes from *Arabidopsis*, rice, and soybean (van der Graaff et al., 2009), *JcWOX* genes of physic nut were classified into three clades, termed the WUS, intermediate, and ancient clade (Figure 1). In addition, the WUS clade had the largest members compared to other plants such as rice (van der Graaff et al., 2009), *Arabidopsis* (van der Graaff et al., 2009), poplar (Zhang et al., 2010), and cotton (He et al., 2019). These results show that the WUS clade is highly conserved in physic nut and other plants, confirming previous studies (van der Graaff et al., 2009).

The exon-intron organization can be used as supporting evidence to determine the evolutionary relationships among genes or organisms. The exon-intron splicing arrangement and intron numbers in the *JcWOX* genes were similar to those reported in *Arabidopsis* (van der Graaff et al., 2009), cotton (He et al., 2019), and poplar (Zhang et al., 2010). For example, the members of ancient clade had two introns (Figure 2); similar results are also found in *Arabidopsis* and other plants (van der Graaff et al., 2009). The motif analysis showed that motifs 1 and 2 were uniformly observed in all JcWOX proteins (Supplementary Figure S3), similar to *Arabidopsis* (van der Graaff et al., 2009), cotton (He et al., 2019), rape (Li et al., 2019), and poplar (Zhang et al., 2010). This result indicated that the evolution of WOX transcription factors was conserved in plant development. Taken together, WOX proteins in the same group shared similar gene structures and conserved motifs, showing that the classification of these proteins involved in this study and the evolutionary relationship between them were reliable.

The expression profile of genes is associated with their functions. We therefore detected the transcript abundance of 12 *JcWOX* genes sequencing-based transcriptome data. The results displayed that *JcWOX5* was preferentially expressed in roots (Figure 4), and its rice homolog *OsWOX3A* was found to participate in the development of lateral roots (Cho et al., 2013), displaying that the *JcWOX5* gene may play an important role in physic nut root development. *JcWOX8* was detected to have high expression levels in all tissues tested (Figure 4), suggesting that the *JcWOX8* gene may be involved in the fundamental elements of plant growth and development processes. *JcWOX3*, *7*, and *12* had a high expression in seeds (Figure 4), indicating that these genes may have a function in regulating seed development. In addition, some *JcWOX* genes had weak or no expression in the tissues tested (Figure 4). A likely explanation is that the *WOX* genes are usually expressed at some specific locations, such as embryos or quiescent centers in roots.

A growing body of research shows that *WOX* genes participated in response to various abiotic stresses in different plant species (Yang et al., 2017; Hao et al., 2019). For example, in soybean, drought or salt stress can up-regulate or down-regulate the transcriptional abundance of some *WOX* genes (Hao et al., 2019). In cotton, some *WOX* genes can also be induced or suppressed by abiotic stress such as drought (Yang et al., 2017). In rice, *OsWOX13*-overexpressing plants have a higher tolerance under drought stress (Minh-Thu et al., 2018). However, no *WOX* genes that responded to drought and salinity have been reported in physic nut. Thus, we tested the expression profiles of *JcWOX* genes exposed to drought and salinity and identified five *JcWOX* genes that were clearly involved in the response to drought and salinity stress (Figure 5). For instance, salinity stress induced *JcWOX1* and *8* genes expression compared with the control, while *JcWOX5* and *6* were down-regulated under drought stress treatment (Figure 5). In summary, our findings suggest that *WOX* gene products may have important functions in regulating physic nut in response to abiotic stress, and provide important reference information for the development and utilization of subsequent functional genes; their role requires further genetic verification.

Drought severely limits crop growth and final yield, so we urgently need to elucidate the molecular mechanism of plant response to drought stress and identify ways to weaken this damage. We noticed that *JcWOX5*, a member from the WOX family, significantly down-regulated expression by drought stress, and to explore its function, we detected the effect of this gene in transgenic rice. *JcWOX5*-overexpressing plants increased sensitivity to drought stress, exhibited a higher rate of water loss, more severe leaf curl, and lower survival rate compared to WT (wild-type) crops (Figures 8A–C). Overall, our data suggest that *JcWOX5* is involved in drought stress response in transgenic rice.

Relative conductivity can be used as a physiological factor to measure the damage of abiotic stress to plant cell membranes. Our data indicated that when subjected to drought stress, *WOX5*-overexpressing plants had a higher relative conductivity compared to wild-type plants (Figure 8D), indicating that drought stress damaged the cell membrane of transgenic plants more than wild-type. Proline is a common compatible osmolyte that protects cell membrane systems from the detrimental effects of drought and salinity stresses, and is commonly used as a marker to assess the extent of drought damage to cell membranes (Yuan et al., 2016). Proline content was increased in wild-type and transgenic plants with *JcWOX5* under drought stress, but the accumulation was obviously higher in wild-type plants than in *JcWOX5*-overexpressing plants (Figure 8E). The finding displayed that drought stress had more damage to cell members of transgenic plants than that of wild-type plants. Additionally, drought stress causes massive accumulation of MDA by promoting lipid peroxidation, so MDA can also be used as an indicator of impairment caused by drought stress (Sathiyaraj et al., 2011). In this study, transgenic plants with *JcWOX5* had a higher MDA content than wild-type plants exposed to drought stress (Figure 8G), displaying that drought stress had more damage to transgenic plants than wild-type plants. Collectively, these results indicate that up-regulated expression of the *JcWOX5* gene can increase transgenic plants’ sensitivity to drought stress, and this biological function is likely to be achieved by increasing the relative conductivity, reducing the content of proline and CAT activity, and increasing the content of MDA.

Various stresses, including drought and high salinity, can give rise to the expression levels of abiotic-stress-related genes, which further protect plants from abiotic stress (Nguyen et al., 2018). *OsLEA3* has been demonstrated to confer tolerance to drought stress to transgenic rice (Hu, 2008). Previous research has shown that increasing the expression of the *OsP5CS* gene can accumulate more proline, and *OsP5CS-*overexpressing plants have an enhanced tolerance to abiotic stress (Hien et al., 2003). Abiotic stress can upregulate the transcription levels of *OsSNAC1* and *OsDREB2A* genes, and overexpression of these genes in crops increases the resistance of transgenic crops to drought stress (Cui et al., 2011; Liu et al., 2014). In this study, expression of *OsLEA3*, *OsSNAC1*, *OsDREB2A*, and *OsP5CS* was obviously lower in *JcWOX5*-overexpressing plants than wild-type plants exposed to drought stress. However, there was no significant difference in the transcription levels of these genes when exposed to non-stress conditions (Figure 8H), although the gene expression was driven by a constitutive promoter. Similar results were also found in nNOS-overexpressing plants (Cai et al., 2015). A likely reason is that other regulators, which respond to stress, are required to induce *JcWOX5*-dependent, stress-related genes when exposed to drought stress. Our finding strongly suggests that *JcWOX5* negatively regulates drought response in transgenic plants at least partly due to lower expression of these stress-related genes.

## Conclusion

In this study, we identified 12 *WOX* genes in physic nut genome. Phylogenetic analysis identified three groups, named the WUS, intermediate, and ancient clade, which was further supported by their gene structures and conserved motifs. Transgenic expression in rice of one of the genes (*JcWOX5*) increased transgenic plants’ sensitivity to drought stress, supporting the hypothesis that some members of the WOX family participated in the regulation of physic nut response to abiotic stress. These findings can provide some basis for the prediction of *JcWOX* genes function in plant stress resistance and development, and the results of a comprehensive analysis of the WOX family will be useful in screening genes for further functional studies and genetic improvement of important stress-resistant varieties in physic nut.

## Data Availability Statement

Publicly available datasets were analyzed in this study. Drought stress raw data for the SRA (sequence read archive) at NCBI was PRJNA257901, whereas salt stress was PRJNA244896.

## Author Contributions

YT conceived and designed the experiments and wrote the manuscript. HL, YaG, SL, CX, EP, JL, RH, QS, YD, YuG, and HZ performed the experiments and analyzed the data. XB revised the manuscript. All authors had read and approved the final manuscript.

## Conflict of Interest

The authors declare that the research was conducted in the absence of any commercial or financial relationships that could be construed as a potential conflict of interest.

## Footnotes

1 http://www.Arabidopsis.org/

2 http://www.phytozome.net/

3 http://www.ncbi.nlm.nih.gov/

4 http://gsds.cbi.pku.edu.cn/

## Supplementary Material

FIGURE S1 Sequence alignments of HD domains of representative WOX proteins in physic nut carried out using the DNAMAN6.0 program. The complete conserved amino acid residues of WOX were labeled with an asterisk.

FIGURE S2 Neighbor-joining unrooted tree of WOX proteins from physic nut and *Arabidopsis*. Bootstrap values were calculated for 1000 replicates, and values are indicated at the corresponding nodes.

FIGURE S3 Motifs analysis of WOX proteins in physic nut. The differently colored boxes represent different motifs.

TABLE S1 Primers used in this study.

TABLE S2 Summary of *JcWOX* genes encoding WOX proteins in physic nut.

TABLE S3 Sequences of proteins encoded by *WOX* genes in *Arabidopsis*, rice, soybean, and poplar.

TABLE S4 Signal strength values for the expression of 12 *JcWOX* genes in tested tissues (root, stem cortex, leaf, and seed) based on RNA-seq.

## References

[B1] Abel S. Theologis A. (1998). Transient gene expression in protoplasts of *Arabidopsis thaliana*. *Methods Mol. Biol.* 82 209–217. 10.1385/0-89603-391-0 9664427

[B2] Butt H. I. Yang Z. Gong Q. Chen E. Wang X. Zhao G. (2017). GaMYB85, an R2R3 MYB gene, in transgenic Arabidopsis plays an important role in drought tolerance. *BMC Plant Biol.* 17:142. 10.1186/s12870-017-1078-3 28830364 PMC5568319

[B3] Cai W. Liu W. Wang S. Fu Z. W. Han T. Lu Y. (2015). Overexpression of rat neurons nitric oxide synthase in rice enhances drought and salt tolerance. *PLoS One* 10:e0131599. 10.1371/journal.pone.0131599 26121399 PMC4485468

[B4] Cannon S. B. Mitra A. Baumgarten A. Young N. D. May G. (2004). The roles of segmental and tandem gene duplication in the evolution of large gene families in *Arabidopsis thaliana*. *BMC Plant Biol.* 4:10. 10.1186/1471-2229-4-10 15171794 PMC446195

[B5] Cho S. H. Yoo S. C. Zhang H. Pandeya D. Koh H. J. Hwang J. Y. (2013). The rice narrow leaf2 and narrow leaf3 loci encode WUSCHEL-related homeobox 3A (OsWOX3A) and function in leaf, spikelet, tiller and lateral root development. *New Phytol.* 198 1071–1084. 10.1111/nph.12231 23551229

[B6] Costanzo E. Trehin C. Vandenbussche M. (2014). The role of WOX genes in flower development. *Ann. Bot.* 114 1545–1553. 10.1093/aob/mcu123 24973416 PMC4204783

[B7] Cui M. Zhang W. Zhang Q. Xu Z. Zhu Z. Duan F. (2011). Induced over-expression of the transcription factor OsDREB2A improves drought tolerance in rice. *Plant Physiol. Biochem.* 49 1384–1391. 10.1016/j.plaphy.2011.09.012 22078375

[B8] Duncan D. B. (1955). Multiple range and multiple F tests. *Biometrics* 11 1–42.

[B9] Gao B. Wen C. Fan L. Kou Y. Ma N. Zhao L. (2014). A rosa canina WUSCHEL-related homeobox gene, RcWOX1, is involved in auxin-induced rhizoid formation. *Plant Mol. Biol.* 86 671–679. 10.1007/s11103-014-0255-0 25301174

[B10] Gonzali S. Novi G. Loreti E. Paolicchi F. Poggi A. Alpi A. (2005). A turanose-insensitive mutant suggests a role for WOX5 in auxin homeostasis in *Arabidopsis thaliana*. *Plant J.* 44 633–645. 10.1111/j.1365-313X.2005.02555.x 16262712

[B11] Hao Q. Zhang L. Yang Y. Shan Z. Zhou X. (2019). Genome-wide analysis of the WOX gene family and function exploration of GmWOX18 in soybean. *Plants* 8:215. 10.3390/plants8070215 31373320 PMC6681341

[B12] He P. Zhang Y. Liu H. Yuan Y. Wang C. Yu J. (2019). Comprehensive analysis of WOX genes uncovers that WOX13 is involved in phytohormone-mediated fiber development in cotton. *BMC Plant Biol.* 19:312. 10.1186/s12870-019-1892-x 31307379 PMC6632001

[B13] Hien D. T. Jacobs M. Angenon G. Hermans C. Thu T. T. Le V. S. (2003). Proline accumulation and Δ 1 -pyrroline-5-carboxylate synthetase gene properties in three rice cultivars differing in salinity and drought tolerance. *Plant Sci.* 165 1059–1068. 10.1016/s0168-9452(03)00301-7

[B14] Hu T. (2008). OsLEA3, a late embryogenesis abundant protein gene from rice, confers tolerance to water deficit and salt stress to transgenic rice. *Russ. J. Plant Physl.* 55 530–537. 10.1134/s1021443708040158 PMC157716 12226181

[B15] Ishiwata A. Ozawa M. Nagasaki H. Kato M. Noda Y. Yamaguchi T. (2013). Two WUSCHEL-related homeobox genes, narrow leaf2 and narrow leaf3, control leaf width in rice. *Plant Cell Physiol.* 54 779–792. 10.1093/pcp/pct032 23420902

[B16] Jiang J. Ma S. Ye N. Jiang M. Cao J. Zhang J. (2017). WRKY transcription factors in plant responses to stresses. *J. Integr. Plant Biol.* 59 86–101. 10.1111/jipb.12513 27995748

[B17] Li M. Wang R. Liu Z. Wu X. Wang J. (2019). Genome-wide identification and analysis of the WUSCHEL-related homeobox (WOX) gene family in allotetraploid Brassica napus reveals changes in WOX genes during polyploidization. *BMC Genomics* 20:317. 10.1186/s12864-019-5684-3 31023229 PMC6482515

[B18] Liu G. Li X. Jin S. Liu X. Zhu L. Nie Y. (2014). Overexpression of rice NAC gene SNAC1 improves drought and salt tolerance by enhancing root development and reducing transpiration rate in transgenic cotton. *PLoS One* 9:e86895. 10.1371/journal.pone.0086895 24489802 PMC3904958

[B19] Minh-Thu P. T. Kim J. S. Chae S. Jun K. M. Lee G. S. Kim D. E. (2018). A WUSCHEL homeobox transcription factor, OsWOX13, enhances drought tolerance and triggers early flowering in rice. *Mol. Cells* 41 781–798. 10.14348/molcells.2018.0203 30078233 PMC6125423

[B20] Nguyen H. C. Lin K. Ho S. L. Chiang C. M. Yang C. (2018). Enhancing the abiotic stress tolerance of plants: from chemical treatment to biotechnological approaches. *Physiol. Plant.* 164 452–466. 10.1111/ppl.12812 30054915

[B21] Openshaw K. (2000). A review of Jatropha curcas: an oil plant of unfulfilled promise. *Biomass Bioenerg.* 19 1–15. 10.1016/S0961-9534(00)00019-2

[B22] Park S. O. Zheng Z. Oppenheimer D. G. Hauser B. A. (2005). The PRETTY FEW SEEDS2 gene encodes an Arabidopsis homeodomain protein that regulates ovule development. *Development* 132 841–849. 10.1242/dev.01654 15659481

[B23] Romera-Branchat M. Ripoll J. J. Yanofsky M. F. Pelaz S. (2013). The WOX13 homeobox gene promotes replum formation in the *Arabidopsis thaliana* fruit. *Plant J.* 73 37–49. 10.1111/tpj.12010 22946675

[B24] Sakakibara K. Reisewitz P. Aoyama T. Friedrich T. Ando S. Sato Y. (2014). WOX13-like genes are required for reprogramming of leaf and protoplast cells into stem cells in the moss physcomitrella patens. *Development* 141 1660–1670. 10.1242/dev.097444 24715456

[B25] Sathiyaraj G. Lee O. R. Parvin S. Khorolragchaa A. Kim Y. J. Yang D. (2011). Transcript profiling of antioxidant genes during biotic and abiotic stresses in Panax ginseng C. A. Meyer. *Mol. Biol. Rep.* 38 2761–2769. 10.1007/s11033-010-0421-7 21086178

[B26] Tang Y. Bao X. Wang S. Liu Y. Tan J. Yang M. (2019a). A physic nut stress-responsive HD-Zip transcription factor, JcHDZ07, confers enhanced sensitivity to salinity stress in transgenic Arabidopsis. *Front. Plant Sci.* 10:942. 10.3389/fpls.2019.00942 31379913 PMC6652468

[B27] Tang Y. Wang J. Bao X. Liang M. Lou H. Zhao J. (2019b). Genome-wide identification and expression profile of HD-ZIP genes in physic nut and functional analysis of the JcHDZ16 gene in transgenic rice. *BMC Plant Biol.* 19:298. 10.1186/s12870-019-1920-x 31286900 PMC6615155

[B28] Ueda M. Zhang Z. Laux T. (2011). Transcriptional activation of Arabidopsis axis patterning genes WOX8/9 links zygote polarity to embryo development. *Dev. Cell* 20 264–270. 10.1016/j.devcel.2011.01.009 21316593

[B29] van der Graaff E. Laux T. Rensing S. A. (2009). The WUS homeobox-containing (WOX) protein family. *Genome Biol.* 10:248. 10.1186/gb-2009-10-12-248 20067590 PMC2812940

[B30] Wang H. Niu L. Fu C. Meng Y. Sang D. Yin P. (2017). Overexpression of the WOX gene STENOFOLIA improves biomass yield and sugar release in transgenic grasses and display altered cytokinin homeostasis. *PLoS Genet.* 13:e1006649. 10.1371/journal.pgen.1006649 28264034 PMC5358894

[B31] Wu P. Zhou C. Cheng S. Wu Z. Lu W. Han J. (2015). Integrated genome sequence and linkage map of physic nut (*Jatropha curcas* L.), a biodiesel plant. *Plant J.* 81 810–821. 10.1111/tpj.12761 25603894

[B32] Xie Z. Nolan T. M. Jiang H. Yin Y. (2019). AP2/ERF transcription factor regulatory networks in hormone and abiotic stress responses in Arabidopsis. *Front. Plant Sci.* 10:228. 10.3389/fpls.2019.00228 30873200 PMC6403161

[B33] Xu M. Xie W. Huang M. (2015). Two WUSCHEL-related HOMEOBOX genes, PeWOX11a and PeWOX11b, are involved in adventitious root formation of poplar. *Physiol. Plant* 155 446–456. 10.1111/ppl.12349 25998748

[B34] Yang Z. Qian G. Qin W. Yang Z. Li F. (2017). Genome-wide analysis of WOX genes in upland cotton and their expression pattern under different stresses. *BMC Plant Biol.* 17:113. 10.1186/s12870-017-1065-8 28683794 PMC5501002

[B35] Yuan X. Wang H. Cai J. Bi Y. Li D. Song F. (2019). Rice NAC transcription factor ONAC066 functions as a positive regulator of drought and oxidative stress response. *BMC Plant Biol.* 19:278. 10.1186/s12870-019-1883-y 31238869 PMC6593515

[B36] Yuan Y. Fang L. Karungo S. K. Zhang L. Gao Y. Li S. (2016). Overexpression of VaPAT1, a GRAS transcription factor from *Vitis amurensis*, confers abiotic stress tolerance in Arabidopsis. *Plant Cell Rep.* 35 655–666. 10.1007/s00299-015-1910-x 26687967

[B37] Zhang X. Zong J. Liu J. Yin J. Zhang D. (2010). Genome-wide analysis of WOX gene family in rice, sorghum, maize, Arabidopsis and poplar. *J. Integr. Plant Biol.* 52 1016–1026. 10.1111/j.1744-7909.2010.00982.x 20977659

[B38] Zhu J. Shi H. Lee B. H. Damsz B. Cheng S. Stirm V. (2004). An Arabidopsis homeodomain transcription factor gene, HOS9, mediates cold tolerance through a CBF-independent pathway. *Proc. Natl. Acad. Sci. U.S.A.* 101 9873–9878. 10.1073/pnas.0403166101 15205481 PMC470766

