# Supplementary figures and images for: Genome-Wide Identification of the Physic Nut WUSCHEL-Related Homeobox Gene Family and Functional Analysis of the Abiotic Stress Responsive Gene JcWOX5
Source: Front Genet. 2020 Jun 19;11:670. doi: 10.3389/fgene.2020.00670 (PMC7325900; doi:10.3389/fgene.2020.00670)

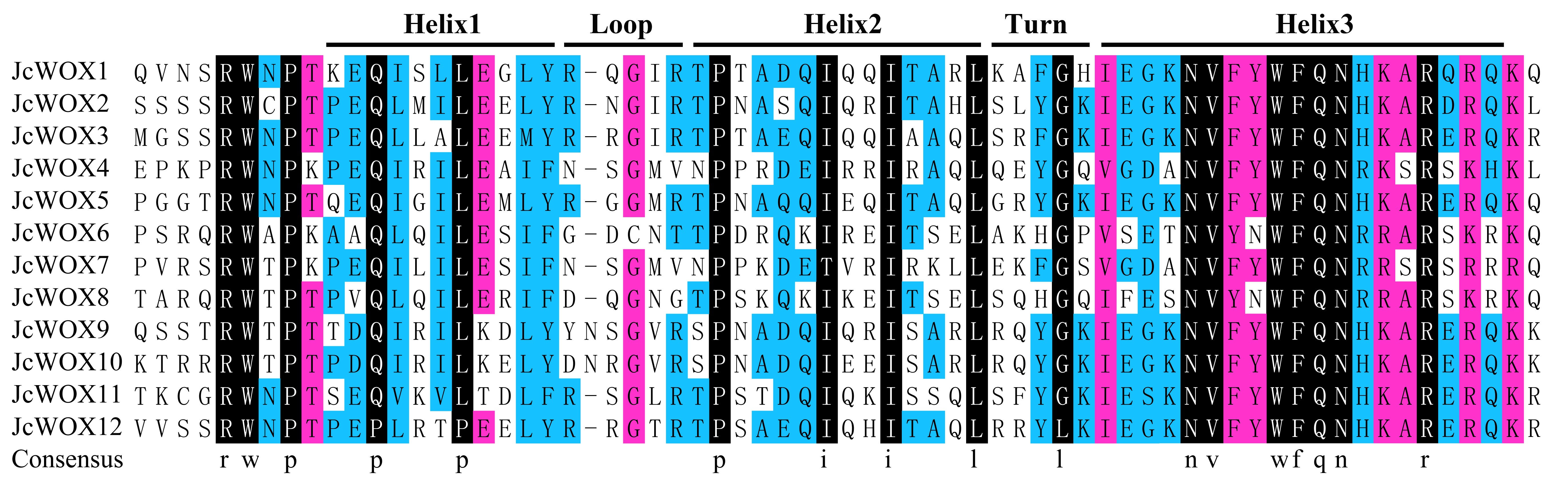

Supplement: FIGURE S1 — Sequence alignments of HD domains of representative WOX proteins in physic nut carried out using the DNAMAN6.0 program. The complete conserved amino acid residues of WOX were labeled with an asterisk. [file Image_1.TIF]

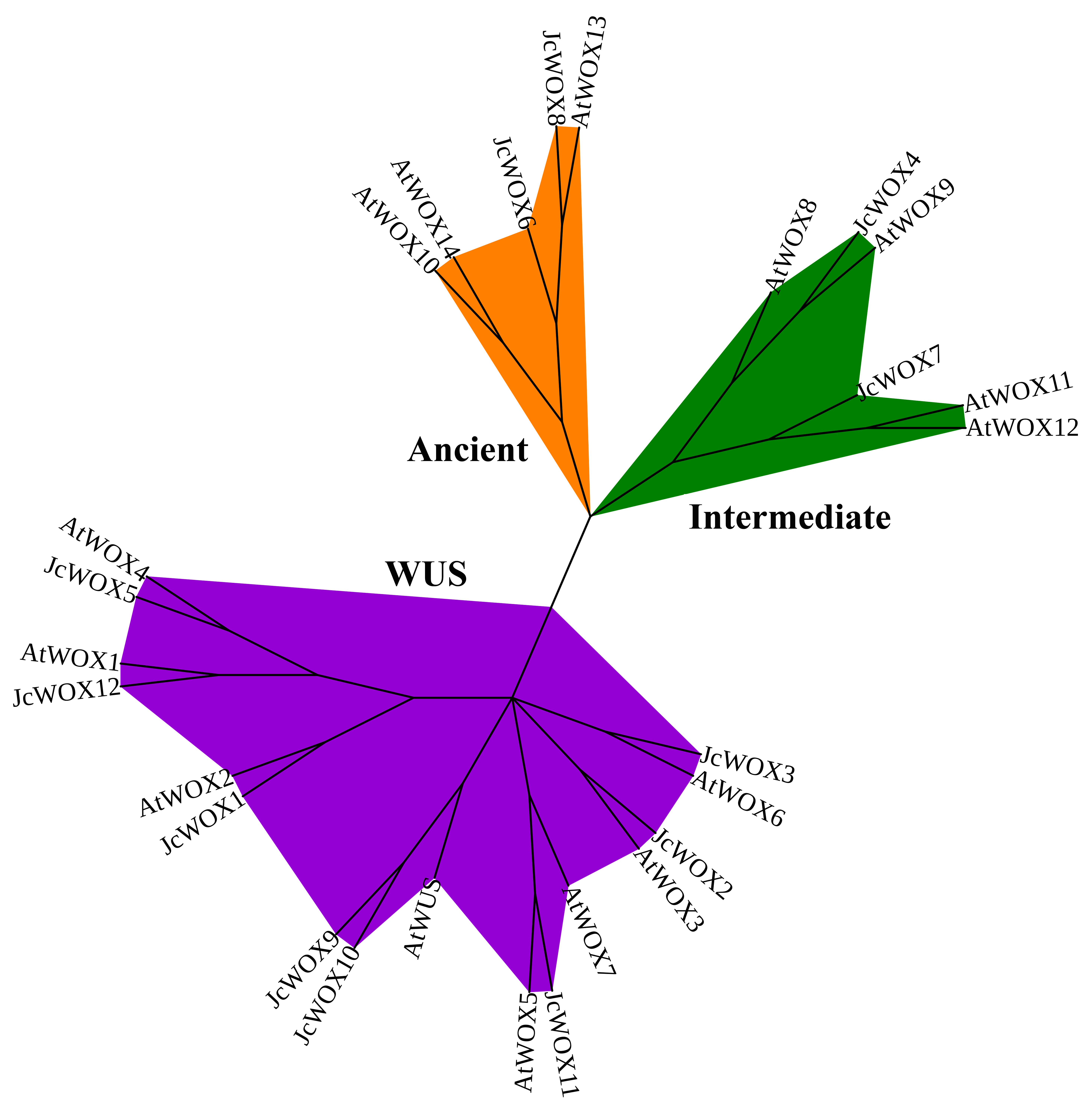

Supplement: FIGURE S2 — Neighbor-joining unrooted tree of WOX proteins from physic nut and Arabidopsis. Bootstrap values were calculated for 1000 replicates, and values are indicated at the corresponding nodes. [file Image_2.TIF]

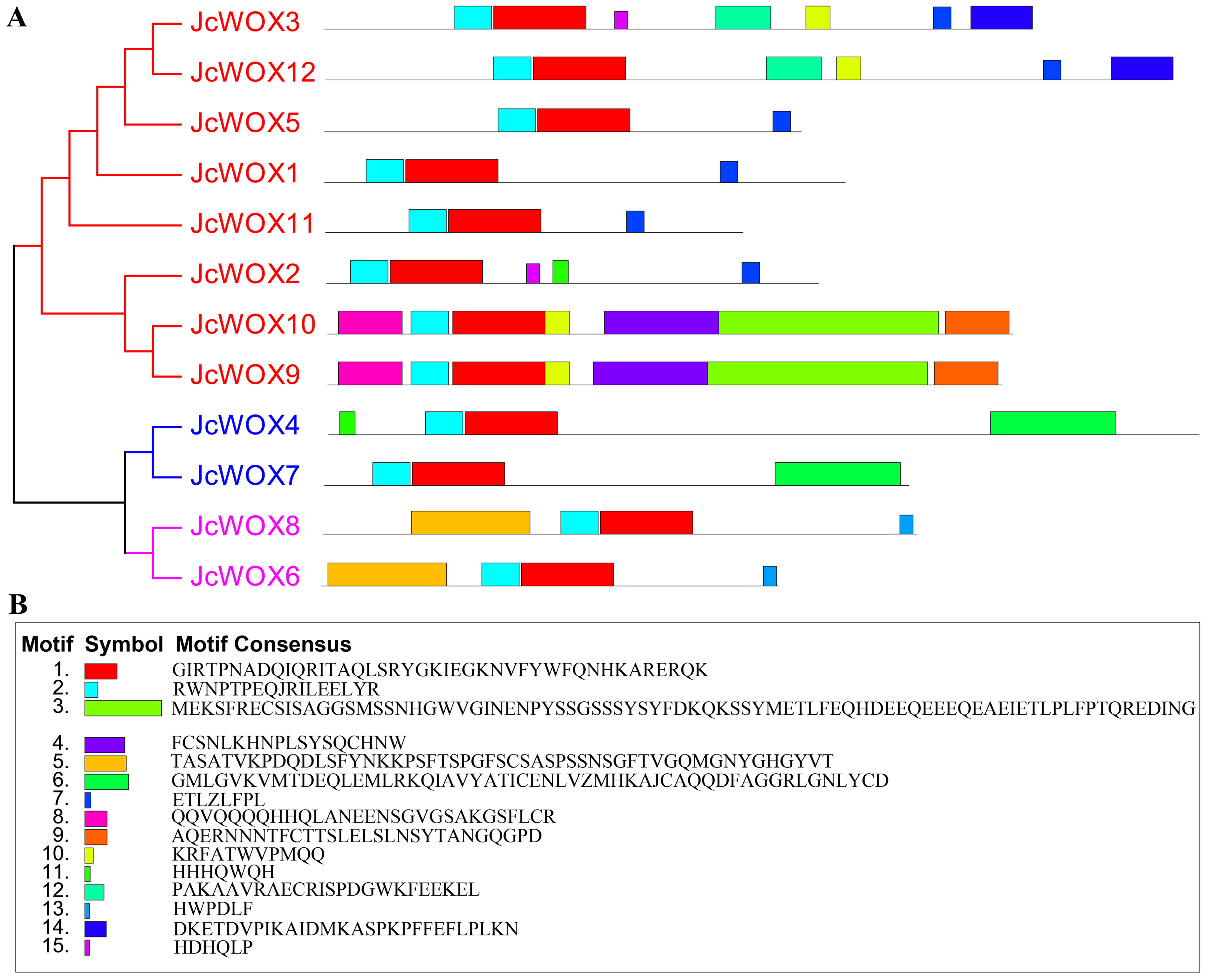

Supplement: FIGURE S3 — Motifs analysis of WOX proteins in physic nut. The differently colored boxes represent different motifs. [file Image_3.TIF]
