# Supplementary material for: Genome-Wide Identification of the Physic Nut WUSCHEL-Related Homeobox Gene Family and Functional Analysis of the Abiotic Stress Responsive Gene JcWOX5
Source: Front Genet. 2020 Jun 19;11:670. doi: 10.3389/fgene.2020.00670 (PMC7325900; doi:10.3389/fgene.2020.00670)
Supplement: TABLE S4 — Signal strength values for the expression of 12 JcWOX genes in tested tissues (root, stem cortex, leaf, and seed) based on RNA-seq. [file Data_Sheet_1.docx]

>AtWOX3

MSPVASTRWCPTPEQLMILEEMYRSGIRTPNAVQIQQITAHLAFYGRIEGKNVFY

WFQNHKARDRQKLRKKLAKQLHQQQHQLQLQLQQIKPKPISSMISQPVNKNIIDH

HNPYHHHHHNHHHNHHRPYDHMSFDCCSHPSPMCLPHQGTGVGEAPSKVMNEYYC

TKSGAEEILMQKSITGPNSSYGRDWMMMMDMGPRPSYPSSSSSPISCCNMMMSSP

KIPLKTLELFPISSINSKQDSTKL

>AtWOX1

MWTMGYNEGGADSFNGGRKLRPLIPRLTSCPTAAVNTNSDHRFNMAVVTMTAEQN

KRELMMLNSEPQHPPVMVSSRWNPTPDQLRVLEELYRQGTRTPSADHIQQITAQL

RRYGKIEGKNVFYWFQNHKARERQKRRRQMETGHEETVLSTASLVSNHGFDKKDP

PGYKVEQVKNWICSVGCDTQPEKPSRDYHLEEPANIRVEHNARCGGDERRSFLGI

NTTWQMMQLPPSFYSSSHHHHQRNLILNSPTVSSNMSNSNNAVSASKDTVTVSPV

FLRTREATNTETCHRNGDDNKDQEQHEDCSNGELDHQEQTLELFPLRKEGFCSDG

EKDKNISGIHCFYEFLPLKN

>AtWOX4

MKVHEFSNGFSSSWDQHDSTSSLSLSCKRLRPLAPKLSGSPPSPPSSSSGVTSATF

DLKNFIRPDQTGPTKFEHKRDPPHQLETHPGGTRWNPTQEQIGILEMLYKGGMRTP

NAQQIEHITLQLGKYGKIEGKNVFYWFQNHKARERQKQKRNNLISLSCQSSFTTTG

VFNPSVTMKTRTSSSLDIMREPMVEKEELVEENEYKRTCRSWGFENLEIENRRNKN

SSTMATTFNKIIDNVTLELFPLHPEGR

>AtWOX2

MENEVNAGTASSSRWNPTKDQITLLENLYKEGIRTPSADQIQQITGRLRAYGHIEG

KNVFYWFQNHKARQRQKQKQERMAYFNRLLHKTSRFFYPPPCSNVGCVSPYYLQQA

SDHHMNQHGSVYTNDLLHRNNVMIPSGGYEKRTVTQHQKQLSDIRTTAATRMPISP

SSLRFDRFALRDNCYAGEDINVNSSGRKTLPLFPLQPLNASNADGMGSSSFALGSD

SPVDCSSDGAGREQPFIDFFSGGSTSTRFDSNGNGL

>AtWOX5

MSFSVKGRSLRGNNNGGTGTKCGRWNPTVEQLKILTDLFRAGLRTPTTDQIQKIST

ELSFYGKIESKNVFYWFQNHKARERQKRRKISIDFDHHHHQPSTRDVFEISEEDCQ

EEEKVIETLQLFPVNSFEDSNSKVDKMRARGNNQYREYIRETTTTSFSPYSSCGAE

MEHPPPLDLRLSFL

>AtWOX6

MGYISNNNLINYLPLSTTQPPLLLTHCDINGNDHHQLITASSGEHDIDERKNNIPA

AATLRWNPTPEQITTLEELYRSGTRTPTTEQIQQIASKLRKYGRIEGKNVFYWFQN

HKARERLKRRRREGGAIIKPHKDVKDSSSGGHRVDQTKLCPSFPHTNRPQPQHELD

PASYNKDNNANNEDHGTTEESDQRASEVGKYATWRNLVTWSITQQPEEINIDENVN

GEEEETRDNRTLNLFPVREYQEKTGRLIEKTKACNYCYYYEFMPLKN

>AtWOX7

MSSRGFNIKARGLCNNNNGGGGTGAKCGRWNPTVEQVKLLTDLFKAGLRTPSTDQI

QKISMELSFYGKIESKNVFYWFQNHKARERQKCRKISTVKFDHRQDTDLSKPRRDN

VRRHQLPAKG

>AtWOX9

MASSNRHWPSMFKSKPHPHQWQHDINSPLLPSASHRSSPFSSGCEVERSPEPKPRWN

PKPEQIRILEAIFNSGMVNPPREEIRRIRAQLQEYGQVGDANVFYWFQNRKSRSKHK

LRLLHNHSKHSLPQTQPQPQPQPSASSSSSSSSSSSKSTKPRKSKNKNNTNLSLGGS

QMMGMFPPEPAFLFPVSTVGGFEGITVSSQLGFLSGDMIEQQKPAPTCTGLLLSEIM

NGSVSYGTHHQQHLSEKEVEEMRMKMLQQPQTQICYATTNHQIASYNNNNNNNNIML

HIPPTTSTATTITTSHSLATVPSTSDQLQVQADARIRVFINEMELEVSSGPFNVRDA

FGEEVVLINSAGQPIVTDEYGVALHPLQHGASYYLI

>AtWUS

MEPPQHQHHHHQADQESGNNNNNKSGSGGYTCRQTSTRWTPTTEQIKILKELYYNNA

IRSPTADQIQKITARLRQFGKIEGKNVFYWFQNHKARERQKKRFNGTNMTTPSSSPN

SVMMAANDHYHPLLHHHHGVPMQRPANSVNVKLNQDHHLYHHNKPYPSFNNGNLNHA

SSGTECGVVNASNGYMSSHVYGSMEQDCSMNYNNVGGGWANMDHHYSSAPYNFFDRA

KPLFGLEGHQEEEECGGDAYLEHRRTLPLFPMHGEDHINGGSGAIWKYGQSEVRPCA

SLELRLN

>AtWOX8

MSSSNKNWPSMFKSKPCNNNHHHQHEIDTPSYMHYSNCNLSSSFSSDRIPDPKPRWNP

KPEQIRILESIFNSGTINPPREEIQRIRIRLQEYGQIGDANVFYWFQNRKSRAKHKLR

VHHKSPKMSKKDKTVIPSTDADHCFGFVNQETGLYPVQNNELVVTEPAGFLFPVHNDP

SAAQSAFGFGDFVVPVVTEEGMAFSTVNNGVNLETNENFDKIPAINLYGGDGNGGGNC

FPPLTVPLTINQSQEKRDVGLSGGEDVGDNVYPVRMTVFINEMPIEVVSGLFNVKAAF

GNDAVLINSFGQPILTDEFGVTYQPLQNGAIYYLI

>AtWOX11

MDQEQTPHSPTRHSRSPPSSASGSTSAEPVRSRWSPKPEQILILESIFHSGMVNPPKE

ETVRIRKMLEKFGAVGDANVFYWFQNRRSRSRRRQRQLQAAAAAADATTNTCDQTMMV

SNSLPHHSGSDLGFGGCSTSSNYLFGGSSQVPSFFLGLSSSPSSCSSSSSTSSSASSS

SSYGGGCDNQSNSGMENLLTMSGQMSYHEATHHHYQNHSSNVTSILCPSDQNSNFQYQ

QGAITVFINGVPTEVTRGGIDMKATFGEDLVLVHSSGVPLPTDEFGFLMHSLQHGEAY

FLVPRQT

>AtWOX10

MEQESLNGRYGSRVMTDEQMETLRKQIAIYAVLCDQLVFLHNSLSSVPLLSSGMNPMR

GEYFDPMVASSSAHGMSTRPRWTPTTTQLQILENIYKEGSGTPNPRRIKEITMELSEH

GQIMEKNVYHWFQNRRARSKRKQPPTTTITSSQADDAAVTTTEERGRCGDDSGGFESY

EHILFPSPDLGIEHLLNRDKFID

>AtWOX12

MNQEGASHSPSSTSTEPVRARWSPKPEQILILESIFNSGTVNPPKDETVRIRKMLEKF

GAVGDANVFYWFQNRRSRSRRRHRQLLAATTAAATSIGAEDHQHMTAMSMHQYPCSNN

EIDLGFGSCSNLSANYFLNGSSSSQIPSFFLGLSSSSGGCENNNGMENLFKMYGHESD

HNHQQQHHSSNAASVLNPSDQNSNSQYEQEGFMTVFINGVPMEVTKGAIDMKTMFGDD

SVLLHSSGLPLPTDEFGFLMHSLQHGQTYFLVPRQT

>AtWOX13

MMEWDNQLQPNNHHSSNLQGIDVNGGSGAGGGMYVKVMTDEQYETLRKQIAIYGTICE

RLVEMHKTLTAQQDLAGGRMGGLYADPMMSSLGHKMTARQRWTPTPVQLQILERIFDQ

GTGTPSKQKIKDITEELSQHGQIAEQNVYNWFQNRRARSKRKQHGGGSSGNNNGESEV

ETEVEALNEKRVVRPESLLGLPDGNSNNNGLGTTTATTTAPRPEDLCFQSPEISSDLH

LLDVLSNPRDEHLVGKMGLAESYNLYDHVEDYGMSG

>AtWOX14

MVKKKKEKEKSKEIEEMDREIQNGAYSGRVMTEEQMEILRKQIAVYAVICDQLVLLHN

SLSSYHPLSSGVRPMVGGYFDPMGASSSSHRISTRHRWTPTSTQLQILESIYDEGSGT

PNRRRIREIATELSEHGQITETNVYNWFQNRRARSKRKQPQTTTANGQADDVAVTTEE

RRSCGDSGGLESYEHILFPSPDLGIEHLLSIGKFMET

>Os11g01130

MPQTPSTRWCPTPEQLMILEEMYRSGVRTPNAAEIQQITAHLAYYGRIEGKNVFYWFQNH

KARERQRLRRRLCARHQQQPSPPSSTVPPAPTAAAAGAVVQVHPAVMQLHHHHHHHHPYA

AAAAAQSHHLQQQQQQQAEWPAAVDYCSTASASASATAADMAIPPCCRPLKTLELFPTKS

TSGGLKEDCCSSSKSSSCSTSTN

>Os12g01120

MPQTPSTRWCPTPEQLMILEEMYRSGVRTPNAAEIQQITAHLAYYGRIEGKNVFYWFQNH

KARERQRLRRRLCARHQQQPSPPSSTVPPAPTAAAAGAVVQVHPAVMQLHHHHHHHHPYA

AAAAAQSHHLQQQQQQQAEWPAAVDYCSTASASASATAADMAIPPCCRPLKTLELFPTKS

TSGGLKEDCCSSSKSSSCSTSTN

>Os05g02730

MAPAVQQQQSGGGGGSTGAAAVGSTTRWCPTPEQLMMLEEMYRGGLRTPNAAQIQQITAH

LSTYGRIEGKNVFYWFQNHKARDRQKLRRRLCISHHLLSCAHYYHHHLAAAAAVVPPPQL

LPPLHPSSSSSSCGGGLIDHANSLLSPTSATTPTSAAAAAAAAAYTTSYYYPFTAAAAPP

PPRTSPAASPLFHYNQGGGGVVLPAAEAIGRSSSSSDYSLGKLVDNFGVALEETFPAQPQ

QPATTMAMTAVVDTTAVAAAAGGFCRPLKTLDLFPGGLKEEQHDVV

>Os04g55590

MRLHHLHVAYLDHKASSSSSSPAPPSISPSSIPGSAAFPAFSFKCLRPLAPKISLPEPRK

MIAPPDFVVPRARNASKLLNYTVQVPAAGTTRWNPSAEQIKVLEMLYRGGMRTPNSVQIE

RITEELGKYGRIEGKNVFYWFQNHKARERQKQKRAALLTLSTLDPSLLPATANETKEAPE

KKEKDVEDGLASCKRRCKAWGDGAGDGDAVVATEAAGGCTDEVTLELFPLHPQGKA

>Os01g62310

METTTTTLGGGGGGRAGGFSDPPSPLSPPLSPASAAAAALANARWTPTKEQIAVLEGLYR

QGLRTPTAEQIQQITARLREHGHIEGKNVFYWFQNHKARQRQKQKQQSFDYFSKLFRRPP

PLPVLHRPLARPFPLAMAPTAMPPPPPPPATTTTAACNAGGVMFRTPSFMPVATNNASYY

PQQQTPLLYPGMEVCPHDKSTAQPPATTTMYLQAPPSSAHLAAAAGRGAAEAEGHGRRGG

GAGGRETLQLFPLQPTFVLPDHKPLRAGSACAAVSPTTPSASASFSWESESSDSPSSEAP

PFYDFFGVHSGGR

>Os04g56780

MDHMQQQQRQQVGGGGGEEVAGRGGVPVCRPSGTRWTPTTEQIKILRELYYSCGIRSPNS

EQIQRIAAMLRQYGRIEGKNVFYWFQNHKARERQKKRLTTLDVTTTTAAAADADASHLAV

LSLSPTAAGATAPSFPGFYVGNGGAVQTDQANVVNWDCTAMAAEKTFLQDYMGVSGVGCA

AGAAPTPWAMTTTTREPETLPLFPVVFVGGDGAHRHAVHGGFPSNFQRWGSAAATSNTIT

VQQHLQQHNFYSSSSSQLHSQDGPAAGTSLELTLSSYYCSCSPYPAGSM

>Os01g63510

MEALSGRVGVKCGRWNPTAEQVKVLTELFRAGLRTPSTEQIQRISTHLSAFGKVESKNVF

YWFQNHKARERHHHKKRRRGASSPDSGSNDDDGRAAAHEGDADLVLQPPESKREARSYGH

HHRLMTCYVRDVVETEAMWERPTREVETLELFPLKSYDLEVDKVRYVRGGGGEQCREISF

FDVAAGRDPPLELRLCSFGL

>Os01g47710

MASSNRHWPSMFRSKHATQPWQTQPDMAGSPPSLLSGSSAGSAGGGGYSLKSSPFSSVGE

ERVPDPKPRWNPRPEQIRILEAIFNSGMVNPPRDEIPRIRMQLQEYGQVGDANVFYWFQN

RKSRSKNKLRSGGTGRAGLGLGGNRASAPAAAHREAVAPSFTPPPPILPAPQPVQPQQQL

VSPVAAPTSSSSSSSDRSSGSSKPARATSTQAMSVTTAMDLLSPLAAACHQQMLYQGQPL

ESPPAPAPKVHGIVPHDEPVFLQWPQSPCLSAVDLGAAILGGQYMHLPVPAPQPPSSPGA

AGMFWGLCNDVQAPNNTGHKSCAWSAGLGQHWCGSADQLGLGKSSAASIATVSRPEEAHD

VDATKHGLLQYGFGITTPQVHVDVTSSAAGVLPPVPSSPSPPNAAVTVASVAATASLTDF

AASAISAGAVANNQFQGLADFGLVAGACSGAGAAAAAAAPEAGSSVAAVVCVSVAGAAPP

LFYPAAHFNVRHYGDEAELLRYRGGSRTEPVPVDESGVTVEPLQQGAVYIVVM

>Os05g48990

MASPNRHWPSMFRSNLACNIQQQQQPDMNGNGSSSSSFLLSPPTAATTGNGKPSLLSSGC

EEGTRNPEPKPRWNPRPEQIRILEGIFNSGMVNPPRDEIRRIRLQLQEYGQVGDANVFYW

FQNRKSRTKNKLRAAGHHHHHGRAAALPRASAPPSTNIVLPSAAAAAPLTPPRRHLLAAT

SSSSSSSDRSSGSSKSVKPAAAALLTSAAIDLFSPAPAPTTQLPACQLYYHSHPTPLARD

DQLITSPESSSLLLQWPASQYMPATELGGVLGSSSHTQTPAAITTHPSTISPSVLLGLCN

EALGQHQQETMDDMMITCSNPSKVFDHHSMDDMSCTDAVSAVNRDDEKARLGLLHYGIGV

TAAANPAPHHHHHHHHLASPVHDAVSAADASTAAMILPFTTTAAATPSNVVATSSALADQ

LQGLLDAGLLQGGAAPPPPSATVVAVSRDDETMCTKTTSYSFPATMHLNVKMFGEAAVLV

RYSGEPVLVDDSGVTVEPLQQGATYYVLVSEEAVH

>Os07g48560

MDGGHSPDRHAAAAAGEPVRSRWTPKPEQILILESIFNSGMVNPPKDETVRIRKLLERFG

AVGDANVFYWFQNRRSRSRRRQRQLQAQAQAAAAAASSGSPPTASSGGLAPGHAGSPASS

LGMFAHGAAGYSSSSSSSWPSSPPSVGMMMGDVDYGGGGDDLFAISRQMGYMDGGGGSSS

SAAAGQHQQQQLYYSCQPATMTVFINGVATEVPRGPIDLRSMFGQDVMLVHSTGALLPAN

EYGILLHSLQMGESYFLVTRSS

>Os03g20910

MEGSSNSPDRQSSGGSPPEERGGGGSGGGGGRSAAGEPVRSRWTPKPEQILILESIFNSG

MVNPPKDETVRIRKLLERFGAVGDANVFYWFQNRRSRSRRRQRQMQAAAAAAAAAASSSS

PSANTSPAAASAATVQVGLPPGAVVHTMAMGGSACQYEQQASSSSSSGSTGGSSLGLFAH

GAGASGAGGYLQASCGASASASSALAPGLMGDVVDSGGSDDLFAISRQMGFVGSPRCSPA

SSPATPSSAATAAQQQFYSCQLPAATITVFINGVPMEMPRGPIDLRAMFGQDVMLVHSTG

ALLPVNDYGILMQSLQIGESYFLVTIHLQALTSWSHRSLSTPISQCSFVFMKAPHF

>Os07g34880

MMALGVPPPPSRAYVSGPLRDDDTFGGDRVRRRRRWLKEQCPAIIVHGGGRRGGVGHRAL

AAGVSKMRLPALNAATHRIPSTSPLSIPQTLTITRDPPYPMLPRSHGHRTGGGGFSLKSS

PFSSVGEERVPDPKPRRNPRPEQIRILEAIFNSGMVNPPRDEIPRIRMQLQEYGQVGDAN

VFYWFQNRKSRSKNKLRSGGTGRAGLGLGGNRASEPPAAATAHREAVAPSFTPPPILPPQ

PVQPQQQLVSPVAAPTSLSSSSSDRSSGSSKPARATLTQAMSVTAAMDLLSPLRRSARPR

QEQRHV

>Os08g14400

MDRTATASWEVMSRRGEQQQQLMMQAPASHNGGSGGGEPARSRWAPKPEQILILESIFNS

GMVNPAKDETARIRRLLERFGAVRDANVFYWFQNRRSRSRRRARQLQQACGAALHQLPSA

AAAAGAGGGGDYYHHHHQPSSSPFLMHGGGGGGVVTSTTAAPAVAASGHFLADEVDGGGD

DDLFAISRQMGLMARHGGGDHHYSSYADSDATQLSYQPTGTIQVFINGVAYDVPSGGALD

MAGTFGRDAMLVHSSGEVLPVDEHGVLINSLQMGECYYLVSKSI

>Os01g60270

MEWDKAKASSGEAVDDRGGGEGGLGYVKVMTDEQMEVLRKQISIYATICEQLVEMHRALT

AQQDSIAGMRLGNLYCDPLMVPGGHKITARQRWTPTPMQLQILENIFDQGNGTPSKQKIK

DITAELSQHGQISETNVYNWFQNRRARSKRKQAALPNNNAESEAEADEESPTDKKPKSDR

PLHQNIAMRDHNSERISEMHHFDTEHEQIRRMMYASNDSSSRSSGSLGQMSFYDNVMSNP

RIDHFLGKVESPGSFPHMRSGESFDMY

>GmWOX1

MKVHQFARGFWEHEPSLTLGCKRLRPLAPKLSNTDTISPPHHPVTTFDLKSFIKPESASR

KLGIGSSDDNTNKRDPSSPQGQAETHIPGGTRWNPTQEQIGILEMLYRGGMRTPNAQQIE

QITAQLSKYGKIEGKNVFYWFQNHKARERQKQKRNNLGLAHSPRTTLTTSPPFSCCVITT

MDTTKRGEVVEREEEDSPLKKCRSWAFEYLEDQREEEHRTLELFPLHPEGR

>GmWOX2

MKVHQFARGFWEHEPSLTLGCKRLRPLAPKLSNTDDTISPPHHHPVTTLDLKSFIKPQSA

SRKLGIGSSSDDNNNNNNKRDPSSPHGQVETHIPGGTRWNPTQEQIGILEMLYRGGMRTP

NAQQIEQITAQLSKYGKIEGKNVFYWFQNHKARERQKQKRNNLGLAHSLRTTPTTIVSHP

FSCSVITTLDTTKRGEIVEREEEDSPLKKCRSWAFEYLEDQREEEHRTLELFPLHPEGRR

RGLF

>GmWOX3

MSPAGSSRWSPTTEQLMILEELYRSGIRTPSASQIQQITTHLSFYGRIEGKNVFYWFQNH

KARDRQKLRRKLTKQLQLQQQQQQQQFQLHHHCQQLNQDHITNHFVGSFGYSTARSTTHD

FSFFNSPSLIFQGGAANTPEQALSCKWNVHNPQSNLVENKEMAFCNYGWTLVDVDNQASS

CCTTRPLKTLDLFPLTTTRINEDCTATPPK

>GmWOX4

MSPAGSPRWSPTTEQLMILEELYRSGIRTPSASQIQQITTHLSFYGRIEGKNVFYWFQNH

KARDRQKLRRKLTKQLQLQQQQQQQLQLHHHCPKLNQDHFTNHFVGNFGYSTLRSTTHDF

PFFNPPSLLFQGGAANTSSEQALSCKWNVHNPQSNLVESKEMAFCNYGWTLVDVDNNQAS

SCCTTRPVKTLDLFPLTTTRISEDCTTPPK

>GmWOX5

MGSMKVHQFTRGLIWEHEPFLTLGCKRLRPLAPKLPNTKTITTPFDLKSFIRPESGPRKP

VSSDDTKKDPPSPQGQIETHPGGTRWNPTQEQIGILEMLYKGGMRTPNAQQIEQITVQLG

KYGKIEGKNVFYWFQNHKARERQKQKRSSLASSHSPRTPTIHSVVTLETTRGEVVERDHE

EDSPYKKKCRRWVFDCLEEQNMSSPCEQEEHRTLELFPLHPEGR

>GmWOX6

MESGNNTNHELEMEMESGGSNGNEGVAASSRWNPTKEQISMLENLYKQGIKTPSAEEIQQ

ITARLRAYGHIEGKNVFYWFQNHKARQRQKQKQETFAYFNRFLHTPQPFFSPPICPNAMC

APYYIPQGQGEIGFYPPHQKVFVPVGFRRSPSEKVVPTGMISSNGPLVYEGMHQMQQRIS

DCSNSHFSHQETLDLFPLHPTGILEGKTTTTTDQVSSLASVVSADSSTDTTSADDINEDD

HASPLNQPFFNFFTTSGQGSY

>GmWOX7

MGSMKVHQFTRGFCWEHEPFLTLGCKRLRPLAPKLPNTQTNTIPTTPSVPFDLKSFIRPE

SGPRKPVSSDDSKKDPPSPQGQVETHPGGTRWNPTQEQIGILEMLYKGGIRTPNAQQIEQ

ITVQLGKYGKIEGKNVFYWFQNHKARERQKQKRNSLAFSHSPRTHTIHSIVTLETTRGEV

VERDHEEDSPYKKKCRRWVFECLEEQNMSSACEQEEHRTLELFPLHPEGR

>GmWOX8

MWMVGYNEGAEFNMADYGFNGRKLRPLMPRPVTSPNNTSNTNSPYLTRIHHGNDFFSQYH

NLASVADQGKREFNPPPVVVSSRWNPTPEQLRALEELYRRGTRTPSAEQIQQITAQLRRF

GKIEGKNVFYWFQNHKARERQKRRRQMESVVAEGHHTRDFDSTLEKKTSAQVGQCLKLNR

PRTGHPLQTAVLLQRNLLQYKGQQKQPLQSVEQMDGSNSMKESYNIEETLWRGMPRGI

>GmWOX9

MFSEMFCTKNNTHVNPLYQLSTILFLPTYLLLYKKSECFTSHGNNSKSIVVITSNNKIKY

SSGIGIDNRIVMESGNNNDEELDMEMEMGGSNVNGNGGVAGSSSRWNPTKEQISMLENLY

KQGIKTPSAEEIQQITARLRAYGHIEGKNVFYWFQNHKARQRQKQKQETFAYFNRFLHTP

QPFFSPPICPNAMCAPYYIPQAQGEIGFYHPHPKVLVPVGFRRSPSEKVVPTGMHQMQQR

ISDCCNSHYSNQETLDLFPLHPTGILEGKTTTTTDQVPSLASVSADSSTDTPSAAASPDH

INEDHDHASPLNQPFFDFFTTSGQGSY

>GmWOX10

MWMVGYNEGGEFNMADYGFNGRKLRPLMPRPVTSPNNTSNTNSPCLSRIHHGNNFFSQYH

NLASVADQGKREFNPPPVVVSSRWNPTPEQLRALEELYRRGTRTPSAEQIQQITAQLRRF

GKIEGKNVFYWFQNHKARERQKRRRQMESAAEGHHTRDFDSTLEKKDLGASRTVFEVDQT

KNWAPSTNCSTLAEESVSIQRAAKAAIAECRTDGWLQFDEGELQHRRNFMERNATWHMMQ

LSCPPPPTVSPHLINTSPITSTTSMATATTVTARLMDPKLIKTHDLSFFTSPNRENGIIH

LSSISTQDDNSVESQTLQLFPTRNADRSSDNINQQKETEVSVSAMNAPSQFFEFLPLKN*

>GmWOX11

MEEGMSEFFSSGVSVGGNSGSATTGTKCGRWNPTTEQVKVLTELFSSGLRTPSTDQIQKI

SNQLSFYGKIESKNVFYWFQNHKARERQKRRKVDKDVIRSENSISINSFTQTDFNQLYQV

SEPERVIETLQLFPLNSFGESESKNMRVHASDQCRDSTMFSYTVGEQMDHPPLDLRLSFM

>GmWOX12

MESHSSDAEAENVRTHSSVSRWSPTKEQIDMLENLYKQGIRTPSTEQIQQITSRLRAYGH

IEGKNVFYWFQNHKARQRQKLMKQQTIAYSNRFLRASHPICQNVACAPYCLQRSGFSFYP

QQSKVLASGGISSTGPLGMQRMFDGMQSSEHPDCNREVLTLFPLHPTGILKEKTTHQVPS

LASTSVVAVDEDGHLGNQPFFNFFTTEPRSRE

>GmWOX13

MEEGMSEFCIRGGNSGSATGTKCGRWNPTTEQVKVLTDLFSSGLRTPSTDQIQKISNQLS

FYGKIESKNVFYWFQNHKARERQKRRKVDNDVIRSENSISINSFTQNFTQLYQVSEPERV

METLQLFPLNSFGESESKNMRVHASDQCRDNTMFSYTVGEQMDHPPLDLRLSFM

>GmWOX14

MDEGLSGFCIRSSSGSVRGKSGTKCGRWNPTTEQVKLLTELFRSGLRTPSTDQIQKISTQ

LSFYGKIESKNVFYWFQNHKARERQKNRKLSFDDHKDLICRQNAPSTQSLAEMYQVSKPD

RVIETLQLFPLNSFGESEPEKLRLRASECRDNNNTMFSYTMGEQMEHPPLDLRLSFL

>GmWOX15

MASSNRHWPSMFKSKPCSPHHQWQHDINASLISTSCHRSPYSSGGGGCEERSPEPKPRWN

PKPEQIRILEAIFNSGMVNPPRDEIRKIRAQLQEYGQVGDANVFYWFQNRKSRSKHKLRH

LQNSSSKNMNHHHHMNVDQQNHTIPTNSSLPQTTTTAPSSSSSSSSEKSSPKELIIPTTK

VFSIGFSDAVPNSPTASVNQTRINDIMLPPPPPVEAFFFPVQQQHDVHHGVTSSSQGFCF

SELSNVVHAQQNNVGPCTSLLLSEIVGHGAASAGASKKDKSQMKIMHQPSLLNFCVTTPT

TATTSTPTTTTTVVVPPITTTSTTVSSPLMPQLQGIGDPGGAARSTVFINDVAFEVAVGP

FNVREAFGDDVVLIHASSGQPVLTNQWGLTLHSLQHGACYYLI

>GmWOX16

MASSNRHWPSMFKSKPCNPHHQWQHDINSSLISTSCHRSPYSSGGGGGCEERSPEPKPRW

NPKPEQIRILEAIFNSGMVNPPRDEIRKIRAQLQEYGQVGDANVFYWFQNRKSRSKHKLR

HLQNSSSKNLNHHHHLVDQNHHTPTSLPQTATTAPSSSSSSSSEKSSPKELIIPTTKVFS

IGFSDVMPNSPTASVNQTYFQTRINESIMLPSPPPPVEAFFFPVQQQHHGVTSSSQGFCF

SELSNVVHQHNSVGPCTSLLLSEIVGHGVASASASKKDKSQVKIMHQPSLLNFCVTTPTT

TAPSTTTTVVVPPITTTSSTTVPSPLMHQLQGIGDPGGAARSMVFINDVAFEVALGPFNV

REAFGDDAVLIHASTGQPVLTNQWGLTLHSLQHGACYYLI

>GmWOX17

MMEPQQQQQQAQGSQQQQQNEDGGSGKGGFLSRQSSTRWTPTNDQIRILKELYYNNGIRS

PSAEQIQRISARLRQYGKIEGKNVFYWFQNHKARERQKKRFTSDHNHNNVPMQRPPTNPS

AAWKPDLADPIHTTKYCNISSTAGISSASSSVEMVTVGQMGNYGYGSVPMEKSFRDCSIS

AGGSSGHVGLINHNLGWVGVDPYNSSTYANFFDKIRPSDQETLEEEAENIGATKIETLPL

FPMHGEDIHGYCNLKSNSYNYDGNGWYHTEEGFKNASRASLELSLNSYTRRSPDYA

>GmWOX18

MMEPQQQQGSQQQQQNEDAGGSGKGGFLSRQSSTRWTPTNDQIRILKDLYYNNGIRSPSA

EQIQRISARLRQYGKIEGKNVFYWFQNHKARERQKKRFTFDHNNNNVPMQQRPPTHPNPS

ASAWKPDPIHTKYSNISSTAGISSASSSSVEMVTVGHMGNYGYGSAPMEKSFRDCSISAG

GSSGHVGINHNLGWVGVDPYSSTYANFFDKIRPTDQEEEAENFGATKIETLPLFPMHGED

IHGYCNLKSNSYNYDGNGWYHTEEGFKNASRASLELSLNSYTRRSPDYD

>GmWOX19

MSDSFGSTFHSSAPNSTLNSHAPPKITTFCSIAQPYCICTHCNHILTFNHHVGNLAEEGT

NSTGSHNNNVQSQPQHSTRWSPTPVQLLVLEELYKQGTKTPSAEQIQQIASQLRQFGKIE

GKNVFYWFQNHKARERQKRRRREMEENNNNNAAASSSSGEGLKETGCGVKETKKWASTSN

CSGHAEESALDIAEKGSNGWTQFEERGSIQVLRRNISEKQAKLQEMEMPCFLPITIAAPT

TTSHRTTTHNTQLLSPQNYNNLSSSLINRESLNYYGGDENADSRTLDLFPHKRDDQDGIS

LAERKSSSMLCASASMDTEITSNQFFEFLPLRN

>GmWOX20

MSDSFGSTFHSSTPNSTLNSHAPPKITTFCSIAQPYCICTHCNHILTFNHHVGNVAEEGT

NSTGSHNNNLQSQQQQSTRWSPTPVQLLVLEELYRQGTKTPSAEQIQQIASQLRQFGKIE

GKNVFYWFQNHKARERQKRRRREMEENNNNNISASSSGEGLKETGCGVKETKKWASTSNC

SGHAEDSAALDIAEKGSNGWTQFEGKDSIQVLMRRNIAEKQAKLQEIMEMPCFPPVTMEA

STPTTSHRRTTHYNTQLLTPQNYNNLPSSVINRESLNYYGGEENAAPRTLDLFPHKRDDQ

HGISLAERKSSMFCASASMDTEVTSNNQFFEFLPLRN

>GmWOX21

MWMMGYEGGEFNMVEHSLITGRKLKPLMPRPMTTSLNNAPTTTTPSLSQIHGNDFLSQYH

YHHLEQNKREFNGAAPVVVSSRWNPTPEQLRALEELYRRGTRTPSAEQIQHITAQLRRFG

NIEGKNVFYWFQNHKARERQKRRRQMESDAETPEKKDLAASRTVFEVERTKNWTPSTNCS

TIAEESVSTQRTAKAVAAESRTDGWLLLDEGELQQRRNVFERNARWHVMQLPCPSPSSAP

VTHLINTPPNASVASSMATTTTTVTTRKMDPNLIKTHDLSFFISPQRENSAIYFSGSSST

SEEDNCVESQTLQLFPLRSGDGISDNMKDNETEISASAMNANNLTPGQFFEFLPMKE

>GmWOX22

MGNDFLSQYRYHHLVAEKNKREFNGATPVVVSSRWNPSPEQLRALEELYRRGTRTPSAEQ

IQHITAQLRRFGNIEGKNVFYWFQNHKARERQKRRRQMESDAEPPEKKDLAASRTVFEVE

RTKNWTPSTNCSTMAEESVSTQRTAKAVAAAESRTDGWLLLDEGELQQRRNLFERNATWH

HVMQLPCPSPSAPVTHLINTTPNASAASSSMTTTTTTVTTTRRRKMDPNLIIKTHDLSFF

ISPLQRENSVIYLSSSSSTSEDDNCVDESQTLQLFPLRSTGDGSSDNMMKDNETEISTSA

MNNANNLTTPGQFFEFLPMKE

>GmWOX23

MSSSNRHWPSMFKSKPCNNPHNQWHHDINSSIVSTGCQRSPYANSGGDERTPEPKPRWNP

KPEQIRILEAIFNSGMVNPPRDEIRKIRVQLQEYGQVGDANVFYWFQNRKSRSKHKLRHF

QNTKNQNNAEAQQQHRVDASSSLSQTTPLSSSSSSSDKSSSKELAYNPNGFSFGFSNVND

VAVPNSPTASVNQTYFHPHNHSDNNLLPQEPFSFTMHNNNGQGFVDNNTITTLGFSVPQF

SSNMMQSQLQCQQNVGSCTSLLLSEIMSHGTFSKKDQDKALKIMHPQLSNFPLTSTPTTI

IAPPISTVLDPSPITQLEGVGEVAAGDRAKCITVFINDVVFEIVMGPFNVRQAFGDEAVL

IHSSGNPVPTDEWGITLHPLHHGACYYLV

>GmWOX24

MESHSTAEDESGWKGSSGAHSSVSRWSPTKEQIDMLENFYKQGIRTPSTEQIQQITSRLR

AYGYIEGKNVFYWFQNHKARQRQKLKQKQQSIAYCNCFLHASHPICQNVVCAPYCLQKSG

FSFYPHQPKVLASVGISSRIETGSFGMLRICDGMQSEHPDYNYSTSNREALTLFPLHPTG

ILEEKTTHHSVDVTDKSFVSIAVDENGHLGNQPCFNFQY

>GmWOX25

MSSSNRHWPNMFKSKPCNNPHNQWQHDINSSIVSTGGYQRSPYASGGEERTPEPKPRWNP

KPEQIRILEAIFNSGMVNPPRDEIRKIRVQLQEYGQVGDANVFYWFQNRKSRSKHKLRHF

QNSMNQNHNAEAQQQQKVDASSLSQTTPPSSSSSSSDKSSSKELAYPIGFSFGFSNVNDV

AVPNSPAASVNQTYFQPHNHIDNNLLPQATEPFSFTMHNNNVQGVVDKNTITTLGFSVPQ

FSSNMMQSQLQCQQNVGPCTSLLLNEIMNYGTLSKKDQDEDKALKITHPQLSFPLTSTPP

TTTIAPSISTVPCPITQLQGVGEVAGDRAKCTVFINGVEFEVVMGPFNVHQAFGDEAVLI

HSSGNPVPTDKRGITLHPLHHGAYYYLV

>GmWOX26

MEDQGQQQQQQHQVEAKSPRQGTERSEAVRSRWTPKPEQILILESIFNSGMVNPPKDETV

RIRKLLEKFGAVGDANVFYWFQNRRSRSRRRQRQMMQQQATATATATVTTFDHPQPQTQT

LVNVGGAIPHDHHTLGNLVVATESSATSTMGFGCSSQSSYGFLGSSSSSSSCGGGVIGGQ

QQGMDGFFSSVSSHQMGFPDHHHTSPASSALYPPLDPNLTYQAGYGGPNISGFITVFING

IATELPKGPIDLKTVFGEDVMLVHSSGVPIPTNEFGFLMHNLQHGDSYFLVSKPT

>GmWOX27

MEDQGQQQQQQEAKSPRQGTERSEAVRSRWTPKPEQILILESIFNSGMVNPPKDETVRIR

RLLEKFGAVGDANVFYWFQNRRSRSRRRQRQMMQQAAAAAAATTIDHPQAQAQTLVGGAI

PLDHTTQGNLVVASSATSTMGFGCSSSPSYAFLGSSSSSCGGGVIGGHQQGMEGFFSVSS

QMGFPDHHTSPASSAFYPPLDPNLTYQAGYGGTNISGFITVFLNGIATELPKGPIDLKTV

FGEDVMLVHSSGVPVPTNEFGFLMQNLRHGESYFLVSKPT

>GmWOX28

MVNVVELQKQLQSGNVNVDANGELMYVKVMTDEQLETLRKQIAVYGTICEQLIEMHRTLS

AQQDLAGVRLGNIYCDPLMTSGGHKITSRQRWTPTPVQLQILERIFDQGNGTPSKEKIKE

ITAELGQHGQISETNVYNWFQNRRARSKRKLQNVAAYSNTESEVETEVDSKDKKTKAEEE

FQSQHNNNISTTSGGAEKLCFQNPQVHSDYLQYLNPADSNKPYSMFQTDDNLKSTRNLSH

VSVFNEVLSNSRSEHIGGKMEVGGSVSYNLFHQTGHFNLAG

>GmWOX29

MVNAMEWQKQRCWQPNRNAEMMCVKVMTDEQLEILRKQIAVYATICEQLIEMHKNISAHQ

DLAGIRLGNMYCDPLLGSGGLKIASRQRWTPTAMQLQILERIFDQGIGTPTKEKIKEITN

ELSQHGQISETNVYNWFQNRRARSKRKQQNVAPSANAESEVETEVDSKDKKTKPDEFQAP

PSVSAGPADNLGFQNHDSLQYLNPESNKPDSVFPSDGTRNFHHVPAFDGLLSNSRSNYLT

GKLETPENYELYQPAGDYNIAG

>GmWOX30

MVNVVELQKQLQRWQQSGNVNVNVNVDANGELMYVKVMTDEQLETLRKQIAVYGTICEQL

IEMHRTLSAQQDLAGVRLGNIYCDQLMTSGGHKITSRQRWTPTPVQLQILERIFDQGNGT

PSKEKIKEITAELGQHGQISETNVYNWFQNRRARSKRRLQNVAPSNTESEVDTEVDSKNK

KTKAEEEFQSQHNITTSGGAEKLCFQNPQVYSDHLHYLNPDSNKPYSMFQSDCNLKSTRN

SSHVSVFNEMLSNSRSEYVGGKMEVGGSVSYNLFHQTGDCNLAG

>GmWOX31

MVNVMEWQKQRWQPNGNAEMMYVKVMTDEQLETLRKQIAVYATICEQLIEMHKNISSHQD

LAGIRLGNMYCDPLLGSGGHKIASRQRWTPSAMQLQILERIFDQGTGTPTKEKIKEITNE

LSQHGQISETNVYNWFQNRRARSKRKQQNVAPSANAESEVETEVDSKDKKTKPDEFQSPP

SVSAVPADDNLGFQNHGSLQYLNPESNKPDSVFPSDASLRSTRNFNHVPVFDGLLSNSTS

DYLTGKMEAPENYDLYQPAGDFNMAG*

>GmWOX32

MEAEHHHQTSNAGGIIGGLYVKVMTDDQMELLRQQISVYATICQQLVEMHKAVTTQQDLA

GLRLGNLYCDPLMACSGHKITARQRWTPTPLQLQVLERIFDEGNGTPSKQKIKDITIELG

QHGQISETNVYNWFQNRRARSKRKQLTPALNVVEPEVETEVEVESPKEKKTRAEGFQVQP

YENSSPHRIKDMYIQSPDIGFDQLMSKIEVAGCYSSYFL

>GmWOX33

MEAETSNGGGIGGLYVKVMTDDQMELLRQQISVYATICEQLVEMHKAVTTQQDLAGLRLG

NLYCDPLMACSGHKITARQRWTPTPLQLQILERIFDEGNGTPSKQKIKDITIELGQHGQI

SETNVYNWFQNRRARSKRKQLTPAPNVVEPEVESPKEKKTRAEGFQVQPYENSSPHRIKD

MYIQSPDIGFDQLLGKIEVASCYSSYFL

>PtWOX1

MGSMKVHQLARGFWEHEPSLTLGCKRLRPLAPKLANTDHSVTSFDLKSFIRPDSGPRKLA

SSDEKKDSPQGETHPGGTRWNPTQEQIGILEMLYRGGMRTPNGQQIEDITAQLSRYGKIE

GKNVFYWFQNHKARERQKQKRNSLGLSHSPRTPSPITIISLDTRVCHCTQQP

>PtWOX2

MGSMKVHQLARGFWEHEPFLTLGFKRLRPLAPKLANTDHSAASFDLKSFIRPDSGPRNLA

SSDEKKDSPQGETHPGGTRWNPTQEQIGILEMLYRGGMRTPNGQQIEDITAQLSRYGKIE

GKNVFYWFQNHKARERQKQKRNSLGLSHSPRTPSPVTIISLDTRGEVEGEEDSPYKRKCR

SWTFECLELEDSRSCREKGDRTLELFPLHPEGR

>PtWOX3

MWMMGYNDSGDFDMPDSFNDRKLKTLVPRPLPSTNNTSTASGHPCPGSRLHSTDFLALNQ

YHLGLASMVDQGIREFNTQPVVMSSRWNPTPEQLRTLEELYRRGTRTPSTDQIQDITAQL

RRYGRIEGKNVFYWFQNHKARERQKRRRQMESDSLDDHQQNGHGVEMFERKEPGASMTGY

EGEQTRNWAPSTNCSTLSEESVSISKATKAAMAEYYRPDGWIEFDEGEIMQHRRNLIERN

ATWEMMPFSCPSPTHLLNTISSATATTIATTSASTQGAATVRTMDPTKLMNAHDLNIFIA

PYIENGYHGARINHFNNSVINEGGEYCRDGNDESQTLQLFPIRSGGNGNNIERINERETE

VSVSATETLNANDFSPCQFFEFLPLRI

>PtWOX4

MWMMGYNDGGEFNISDSFNGRKLRPLVPRPIPSTNNTPTASSPPCLGSRLHNTDFFALNQ

YHLASMADQNKREFNTQPVVMSSRWNPTPEQLRTLEDLYRRGTRTPSTDQIQDITAQLRR

YGRIEGKNVFYWFQNHKARERQKRRRQMESDSFDGHLQNGHGIEIFERKESEASRTGYEG

EQTKNWAPSTNCSTLSEESVSISRATKGAMAEYCRPDGWMQLDEGELQHRRNFIERNATW

EMMQLSCPSPTHQRNTISSTSSTTTMSKQGAAAAKLIKAHDLNVFIAPYRENGHHGALIN

QFNSSVINDGDESRGGTGESQTLQLFPLRSGGDGNNNIESINERESEVSVSAAEALNANN

FAPCQFFEFLPLKH

>PtWOX5

MEERMSGFCITKAGRGGGSGNNCGTGTKCGRWNPTTEQVKLLTDLFRSGLRTPSTDEIQN

ISTQLSFYGKIESKNVFYWFQNHKARERQKRRRVSVDEKDAMIHFTEINHVNEPERVIET

LQLFPLNSFDEAGPEKFRFQANECNEAAAAFSYKFGTEMDHPHLDLRLSFV

>PtWOX6

MASSNRHWPSLFKSKPCNPHHHQWQHDINPSSLMSTGCHRNPYASVPGCEERSPEPKPRW

NPKPEQIRILEAIFNSGMVNPPRDEIRKIRAQLQEYGQVGDANVFYWFQNRKSRSKHKLR

NLQNSKQQITPSTTKPVTASLTAPSSLSSSSEKSSPKVSKRTLSLSSPPFIDASNSPNSS

VSQTYFQAQNEFVSEPFFFPVQQTGGETVAFTQGFCFSELSNVVHVQDHTVGPCPSLLLS

EITNSSASKKANHEERNLKMQPQLSYTATSPVTHSIDLAPPLPLSANTSTVSIQSTISQI

QGLGVSGGNERSTVFINDVAFEVAMGPFNVREAFGDDILLIHSSGQPVLTNEWGITLDSL

QHGALYYLVPLSISEHI

>PtWOX7

MEPQQQQHQNQQQPNEDNNGGAKGNFICRQTSTRWTPTTDQIRILKELYYIKGVRSPNGA

EIQQISARLRKYGKIEGKNVFYWFQNHKARERQKKRFTNDVPTQQRTTLKPEDYYSYKYS

GSNNNPGFSSASSSSNTGAVTVGQADNYGYGSVTMQEKKNWDCSVPAGGESMNNINYGSR

GGIYPYSSSYTVFDQDQEAAEKIETLPLFPMHGEDISTSFNINNVNPDFYYSSWYGSDDY

GNATTSRTSLELSLYSYNGQQQDY

>PtWOX8

MEPHQQQPNEDNNGGAKGNFLCRQTSTRWNPTTDQIRILKELYYIKGVRSPNGAEIQQIS

ARLRKYGKIEGKNVFYWFQNHKARERQKKRLTNEVPMQQRTAWKPEDYYSYKYSNSNNNP

GFSSASSSANTGVVTVGQTDSHGYGSVTMQEKNSWDCSAPAGGSNGAGSGSMSNINYGSG

VDINSHSSSYAVFGQEQEAAAKIETLPLFPMLGEDISSSFNINNINPDFYYSSGCGYGDY

GNDTSSRTSLDLSLYSYNGQPQDY

>PtWOX9

MASSNKHWPSMFKSKPCNPHDHQWQHDINPSSIISTGCHRTPYTSVPGCDERSPEPKPRW

NPKPDQIRILEAIFNSGMVNPPRDEIRKIRVQLQEYGQVGDANVFYWFQNRKSRSKHRLR

NLQNSKQHSSQQQKITSPTTKPVTANLAAPSSSSSSSEKSSPKGSKRTLSLSSPTFIDAS

NSPTSSVNKTYFQAHNEFVPEPFFFHSQQTGGGGTGAFAQGFCFSELSNMVHVQDHTVGP

CSRLLLSEIMNSSASKKVNHEERNLKMQPQLCYTPVSPVTGSIGLAPPLTPSTDTSTFAF

QTTINQIQGLGQSSGTTMLTVFINDVAFEVTMGPFNVREAFGDDVLLIQSSGQPVLTNEC

GVTLQSLQHGAFYYLVPFSMSEHI

>PtWOX10

MDSDDMDVAGSGGAPGNSRWNPTKEQISMLESFYSQGIRTPSTEMIEQITSRLKAYGHIE

GKNVFYWFQNHKARQRQKQKQESMAYINNYLHKVHQPVFAPPCANVVCSPYFPQQSEVMG

FCQQHPKMLLPSNFKMRPRSEARTYAFNGYEPAAPYGYHNRITMNKGERTLVTINHKSSS

DQATLPLFPLHPTGTLEGATSICPVGSTDPAENSTNTPSSSEITTGIEEHSGDCKPFFDF

FYGKDS

>PtWOX11

MDVSSSGGASVNSRWSPTKEQISMLESFYSQGIRTPSTEMIEQIASRLKAYGHIEGKNVF

YWFQNHKARQRQKQKQENMAYINKYLHKAHQPVFAPPCRNVVNSPCYLPKSDIMGLCQQH

QNMLLPGNFKRRSRSETISYAFKGYDQEAVLREYHNHITKNKFERSPVTIDKSSSDQETL

PLFPLHPTGILEGASPIFSHGSTSAENSINTPISSEITHGIGEHSADHKPFFDFFSEKDP

FESSH

>PtWOX12

MWMINGGDSNEPSMNDFFNPKPNTTTLCTYTTATPLTYVGLKHHLAKTSEQSRGRKLKEQ

AEATRSSRWNPTAEQLLALEEKYSCGVRTPTTNQIQQITSELRRFGKIEGKNVFYWFQNH

KARERQKHRQVQQKHNNTDHESSNKMKESGPRRTVLGVDQTNNLAPHSKCSTDHVEGPVS

VNGAAIAESGTHGWSEFEERELQQMKSISLDMHAMWQTMDLSSSTPVHRLTSTMTTTASK

FSSLEEHSSLLRPTKTATHANHDGEIREVQTLQLFPLCSDDGNGANGTNNDRNVPIRTIN

TTFTPSQFFEFLPLKN

>PtWOX13

MEDNQGQDPNSPSNHATERSEPVRSRWTPKPEQILILESIFNSGMVNPPKDETVRIRKLL

EKFGSVGDANVFYWFQNRRSRSRRRQRQMQASLVAGEQTNNQQAQASGGAIQYKGCNTSI

GFANSPSFVQSPSSYLVGSSSSYGVVDEDHGGESLYSFSNQMAFQEVEQTSGVTSILYPS

ETSNLHYQTAGFITVFINGIPTEVPRGPLDIKAMFGQDVVLVHSSGVPVPTNEFGFLMQS

LHHGESYFLVIISGA

>PtWOX14

MEDNQGQDHNSQSNHGTERSEPVRSRWTPKPEQILILESIFNSGMVNPPKNETVRIRKLL

EKFGSVGDANVFYWFQNRRSRSRRRQRQMQASGGTSNGFANSPSSYLVGASSSCGVVGED

HGVESLFSFSNQMGFQEFEQTSGVTSIVCPSETSSLHYQTAGFITIFINGVPTEVPRVPL

DVKAMFGQDVMLVHSSGVPVPTNEFGFLVQILHHGESYFLVNISAV

>PtWOX15

MEERMSGFCTTKAGRGGSSGNNYASGTKCGRWNPTIEQGKLLTDLFRSGVRTPSTDEIQN

ISTRLSFYGKIESKNVFYWFQNHKARERQKRRRVSVDEKDVMIRRDDKFSSARYFTEIGQ

VNEREQVIETLQLFPLKSFDEVESEKFRLQANECNEAAAAFSYKFGTEMDRPQLDLRLSF

L

>PtWOX16

MDWDNNQENHQDSHQNQRECRNGINGTNVNVNGNGGTNMLYVKVMTDEQLETLRKQIAVY

AAICEQLVEMHKTLSAQQDLAGGRLGNLYCDPLMASGGHKITARQRWTPTPVQLQILERI

FDQGNGTPSKQKIKEITSELSQHGQISETNVYNWFQNRRARSKRKQLVASSNNAESEVET

EVDSLNEKKKPEIFHAQQNPPRAEDLCFQSPEISSELHFLGDDHLTGKMGVPGNYNLYDQ

AEDYGMAG

>PtWOX17

MEDGKFQNGGGLGVKVMTDEQMEMLRKQISVYATICEQLVEMHKALSVHQDFAGMRLGNP

YFCDPLLSSSVHKIGSRQRWTPKPAQLEILEQIFKQCNATPGRQKIKDITKELAQHGQIS

ETNVYNWFQNRRARSKRKQSALLPNSGESEVETEIEPFKEKKTKPEDNQPDEDATPVSDH

MYLHSPDIGIDQLVGKMESPGSCIPYWQLEQYDLFG

>PtWOX18

MEEGRFQNGGGLGVKVMTDEQMEMLRKQISVYATICEQLVEMHKALSAQQDFAGMGLGNP

YCDPLLSSAVHKIGSRQRWTPKPAQLQILEQIFEQCNATPGRQKIKDITRELAQHGQISE

TNVYNWFQNRRARSKRKQSAVVPNNGESEMETDIESLKEKKTRAEDSQPDENTTPMADHM

YFNSPDIGFDQLMGKIESPGSCIPYWQMEQYDLFG
